# A hybrid machine learning framework for functional annotation of mitochondrial glutathione transport and metabolism proteins in cancers

**DOI:** 10.1186/s12859-025-06051-1

**Published:** 2025-02-11

**Authors:** Luke Kennedy, Jagdeep K. Sandhu, Mary-Ellen Harper, Miroslava Cuperlovic-Culf

**Affiliations:** 1 Department of Biochemistry, Microbiology and Immunology, Faculty of Medicine, University of Ottawa, 451 Smyth Road, Ottawa, ON K1H 8M5 Canada; 2 Ottawa Institute of Systems Biology, University of Ottawa, 451 Smyth Road, Ottawa, ON K1H 8M5 Canada; 3 Human Health Therapeutics Research Centre, National Research Council Canada, 1200 Montreal Road, Bldg M54, Ottawa, ON K1A 0R6 Canada; 4 Digital Technologies Research Centre, National Research Council Canada, 1200 Montreal Road, Bldg M50, Ottawa, ON K1A 0R6 Canada

**Keywords:** Mitochondria, Glutathione, Machine learning, Cancer, Protein function annotation, Transmembrane transport, SLC25, Multi-omics, Knowledge-based, Gene ontology

## Abstract

**Background:**

Alterations of metabolism, including changes in mitochondrial metabolism as well as glutathione (GSH) metabolism are a well appreciated hallmark of many cancers. Mitochondrial GSH (mGSH) transport is a poorly characterized aspect of GSH metabolism, which we investigate in the context of cancer. Existing functional annotation approaches from machine (ML) or deep learning (DL) models based only on protein sequences, were unable to annotate functions in biological contexts.

**Results:**

We develop a flexible ML framework for functional annotation from diverse feature data. This hybrid ML framework leverages cancer cell line multi-omics data and other biological knowledge data as features, to uncover potential genes involved in mGSH metabolism and membrane transport in cancers. This framework achieves strong performance across functional annotation tasks and several cell line and primary tumor cancer samples. For our application, classification models predict the known mGSH transporter SLC25A39 but not SLC25A40 as being highly probably related to mGSH metabolism in cancers. SLC25A10, SLC25A50, and orphan SLC25A24, SLC25A43 are predicted to be associated with mGSH metabolism in multiple biological contexts and structural analysis of these proteins reveal similarities in potential substrate binding regions to the binding residues of SLC25A39.

**Conclusion:**

These findings have implications for a better understanding of cancer cell metabolism and novel therapeutic targets with respect to GSH metabolism through potential novel functional annotations of genes. The hybrid ML framework proposed here can be applied to other biological function classifications or multi-omics datasets to generate hypotheses in various biological contexts. Code and a tutorial for generating models and predictions in this framework are available at: https://github.com/lkenn012/mGSH_cancerClassifiers.

**Supplementary Information:**

The online version contains supplementary material available at 10.1186/s12859-025-06051-1.

## Background

Glutathione (GSH) is a highly abundant tripeptide antioxidant within cells, crucial for many biological processes with its major role in regulation of reactive oxygen species (ROS). Metabolic changes are one of the hallmarks of cancer [1], with well documented alterations in GSH metabolism foremost among them. These alterations of metabolism appear to benefit cancer cells, aiding in tumor proliferation and survival, through not yet fully understood mechanisms and interactions [2]. Alterations of GSH metabolism are essential for tumor proliferation in several cancers [3, 4] either directly where they mitigate perturbations in redox homeostasis [5–7], or indirectly through ferroptosis [7] and metabolism of chemotherapeutics [7–9].

Central to the contributions of GSH in the metabolic characteristics of cancer cells is mitochondrial GSH (mGSH), which is found at millimolar concentrations in the organelle, at similar levels to those in the cytoplasm. Mitochondria are the primary source of ROS, largely as a byproduct of the oxidative phosphorylation system (OXPHOS), and alterations in redox balance and ROS signaling are central to the role of mitochondria in cancer cell proliferation [9]. Many GSH-utilizing enzymes with altered expression patterns in cancers are found in mitochondria, most notably glutathione peroxidase 4 (GPX4) which is a major regulator of the ferroptosis pathway. Also important are mitochondrial glutathione enzymes such as glutathione S-transferase, which conjugates and detoxifies xenobiotics and the peroxiredoxins, which lower ROS via GSH and have also been shown to promote cancer cell survival [10].

The processes involved in the uptake of GSH into mitochondria are poorly understood. While high concentrations of GSH are present in both mitochondria and the cytosol, the synthetic enzymes are exclusively within the cytosol [11]. SLC25A10 and SLC25A11, also known as the mitochondrial dicarboxylate and oxoglutarate carriers, respectively, were initially proposed as the proteins responsible for GSH transport into mitochondria over 25 years ago [12]. However, detailed functional experiments in lipid vesicle systems in 2014 convincingly showed that no GSH transport was mediated by these two proteins [13].

Recent evidence from two groups demonstrated mGSH transport roles for SLC25A39 and SLC25A40 [14, 15]. Specifically, Wang et al*.* [14] identified the transporters through quantitative proteomics of mitochondria from GSH-depleted HeLa cells. In this work authors have shown that SLC25A39 and SLC25A40 provide essential and sufficient mGSH transport into mitochondria in HeLa cells. Subsequently, Shi et al. [15] leveraged CRISPR screening of gene and environment interactions to demonstrate buffering interactions between SLC25A39 and the mitochondrial iron transporter SLC25A37, revealing SLC25A39 as a candidate and further supported by in vitro metabolomics and GSH transport experiments. The roles of these transporters in neurodegenerative diseases and cancer have recently been explored [16]. However, it remains unclear if the remaining GSH import in SLC25A39 knockouts observed in these studies is facilitated by SLC25A40, which is expressed at roughly one tenth the level of SLC25A39, or if other transport mechanisms exist, such as those for other GSH species [17, 18]. Interactions affecting mGSH metabolism and transport, having secondary effects on transport may also be relevant and are even less well understood.

With recent successes of AlphaFold [19] and RosettaFold [20] in the elucidation of protein structure, there is a promising future for computational biology in the related problem of protein function prediction. However, competitions such as Critical Assessment of Functional Annotation [21] have not yet found a solution for de novo function prediction from sequence. General function annotation models like DeepGO and DeepGOPlus [22, 24] represent major advances for the field. Along with sequence-based methods, models that leverage non-sequence features for function annotation exist [23, 26] as alternative approaches to sequence-based approaches. In addition to the general function prediction models, there are many sequence-based models designed for specific protein feature annotation such as protein–protein interactions [24] and antibody design [25].

Omics-based methods for functional annotation are limited. Recent work by Kunc and Kléma [26] utilize characteristics in co-expression networks constructed from genes to predict shared KEGG pathways between genes. Similarly, Wekesa et al. [27] combine differential gene expression information and knowledge of protein–protein interactions through a neighbor-voting algorithm for prediction of shared functions between yeast proteins. Finally, Wang et al. [28] predict gene–gene interactions by combining co-expression features with prior biological knowledge features (e.g., subcellular localization, homology, Reactome similarity).

The approaches of Wekesa et al. and Wang et al. can be classified as hybrid ML approaches, which aim to capture “the best of both worlds” by combining the predictive powers of data-driven approaches of ML with the interpretability of theory-based models such as mechanistic or knowledge-based models. These types of models have been applied to several domains, most notably in physics-informed ML models [29], and with respect to computational biology, hybrid modeling has been applied through diverse frameworks. Non-ML approaches have been used to uncover biological phenomena, which would otherwise be missed; a recent example integrates behavioral, transcriptomic, and network modeling to reveal the role of brain mitochondria and organization in mouse behavior [30]. Alternatively, ML models have been integrated with mechanistic models of metabolism to determine kinetic parameters and predict downstream metabolic effects [31]. P-net uses a neural network architecture based on biological knowledge of hierarchical gene-pathway-process interactions to predict prostate cancer discovery from gene features, such as methylation and copy number [32]. On the other hand, AlphaFold [19] incorporates evolutionary information through multiple sequence alignments and physical structural constraints to inform its protein structure predictions. These hybrid models retain the predictive power of data-driven methods, often performing better than, or comparable to, standard data-driven models, while increasing the interpretability of predictions that are in-part based on biological knowledge. Performance of hybrid models depends on specific application where increase in accuracy with hybrid models was explored in great detail in Mavaie et al. [33], showing that hybrid approaches can provide a combination of strengths from different methodologies and improve performance.

With this, we sought to develop a ML framework that leverages multi-omics data from human cancer cells and existing knowledge to identify potential genes involved in mGSH transport and potential interacting metabolic processes. We developed several ML classifier models that utilize cancer cell line encyclopedia (CCLE) [34, 35] transcriptomics features to predict gene ontology (GO) annotations for genes of relevant GO terms. Specifically, three classifiers were developed for annotating to: glutathione metabolic process (GO:0006749), mitochondrion (GO:0005739), and transmembrane transporter activity (GO:0022857). Additionally, we developed hybrid models for this task which include prior knowledge as features through MitoCarta [36] scores for mitochondrial localization classification, TrSSP [37] scores for transporter classification, or CCLE GSH & glutathione disulfide (GSSG) metabolomics data for glutathione classification. We find that Random Forest (RF) classifiers perform the best from our models tested, with hybrid models outperforming strictly transcriptomics models.

## Methods

All pre-processing, models and other computational work were conducted using in-house code written in Python (Python Software Foundation. Python Language Reference, version 3.8. Available at www.python.org). All algorithms and methods used in ML model building and training were implemented via Python’s scikit-learn package [38] unless stated otherwise. Figures for evaluations of classifier models were produced via the matplotlib and seaborn libraries [39, 40]; GO enrichment plots were produced using ShinyGO 0.77 [41]; and all other images were created with BioRender.com.

### Data collection & pre-processing

#### Metabolomic & transcriptomic datasets

The Broad Institute’s Cancer Cell Line Encyclopedia (CCLE) provides multi-omics data across over 1000 human cancer cell lines (CCLs). Details of the data experimentation and validation are provided in the original publication [34, 42]. Transcriptomics data was preprocessed in the original work using RSEM quantification of transcripts per million (TPM) method; metabolomics data was quantified using liquid chromatography mass spectrometry and preprocessed using standardized LC–MS peak batch correction and median normalization across metabolites and cell lines. CCLE transcriptomics, and metabolomics data were used in building our models [34, 42]. Raw transcriptomics (1019 cell lines) and metabolomics (225 metabolites, 928 cell line samples) data were downloaded from broadinstitute.org (available at the time of writing via depmap.org/portal/download/).

Data were cleaned and imputed by removing all CCLs and transcripts/metabolites with > 30% missing values or standard deviation values of 0 across samples. The resulting dataset had 49,308 transcripts, and 225 metabolites presented across 878 samples coming from cancer cell lines derived from 23 tissues. Remaining missing values were imputed using the k-nearest neighbors (k-NN) imputation algorithm (using Euclidean distance and k = 5, selected to minimize impact of the data structure on the missing data imputation [43]). For metabolomics features, pairwise Spearman correlations were generated across common CCLs between transcript levels in CCLE transcriptomics data and metabolomics levels for each metabolite of interest (GSH, GSSG, 2-oxoglutarate, glutamate, and carnitine). Non-significant correlations (*p*-value ≥ 0.05, Student’s t-test with 2 degrees of freedom) were set to 0. Spearman rho values were normalized by Fisher transformation [44].

For comparison, transcriptomics data for The Cancer Genome Atlas (TCGA) [45] were also downloaded via the National Cancer Institute’s Genomic Data Commons (https://gdc.cancer.gov/about-data/publications/pancanatlas), containing gene expression data for 20,531 genes from 11,069 primary tumor samples spanning 33 cancer types. Like the CCLE data, expression values are preprocessed using RSEM quantification of TPM values. Relevant TCGA samples were selected for use in classifiers via mapping of TCGA and CCLE samples over 22 common tumor types identified by Yu et al. [46] via the “CCLE_meta.txt” file available at https://github.com/katharineyu/TCGA_CCLE_paper.

Feature space reduction while selecting major variances in transcriptomics was performed using principal component analysis (PCA) on z-score normalized expression values. These principal components (PCs) were used as features in classifier models (further described in “Model development and feature selection process”).

#### Knowledge-based features

Mitochondrial localization scores for genes were based on MitoCarta 3.0 [36] (broadinstitute.org/mitocarta). MitoCarta scores are determined through manual curation following prediction by a Naïve-Bayes model which combines features from several independent domains from features used in our analysis, such as homology, sequence domain, and tandem mass spectrometry of purified mitochondria. CCLE genes were assigned MitoCarta scores according to these data, with 1 indicating mitochondrial localization, and 0 indicating non-mitochondrial localization. For genes that are not assigned scores in MitoCarta, a value of − 1 is assigned.

Transporter activity was based on TrSSP [37] values, obtained from Zhao lab webpage (www.zhaolab.org/TrSSP). TrSSP utilizes support vector machine (SVM) models to predict membrane transport proteins and their substrate classes using primary protein sequence features along with position-specific scoring matrices to predict transport function and specificity. Like MitoCarta scores, TrSSP scores were incorporated into the model as classifications for positive, negative, or no prediction by TrSSP (1, 0, − 1, respectively). For use as features in our classifiers, these knowledge-based categorical scores were transformed to two Boolean features using one-hot encoding. Final models use two vectors to represent the positive and negative classifications, the “no classification” vector is redundant as it is inferred where both other classification vectors are 0.

### mGSH transporter classifier models

#### Training and test gene sets selection

For each GO term classification task, classifier training and test genes were selected based on existing knowledge of gene functions. Gene ontology [47] terms were used to determine genes related to mGSH metabolism: glutathione metabolic process GO:0006749 (65 genes), mitochondrion GO:0005739 (1685 genes), transmembrane transporter activity GO:0022857 (1154 genes). These terms cover all classes of ontology terms: biological process, cellular component, and molecular function, respectively. Annotated genes for each term were identified via AmiGO 2 [48] and those genes annotated based on only low confidence computational or inferred evidence were removed. This left 40 genes for GO:0006749, 781 genes for GO:0005739, and 700 genes for GO:0022857 annotated based on experimental evidence to create the final list of positive class genes for the classifier models. We found that there is little overlap among these gene sets (Supp. Fig. 1a); primarily, common genes are found between mitochondrial and transmembrane transporter activity genes (72 genes), with 5 genes overlapping the mitochondrial and glutathione metabolic process sets and only one gene found in both transmembrane transporter activity and glutathione metabolic process genes. There are no genes annotated by all three GO terms. AmiGO annotations based on sequence similarities were retained along with the experimental AmiGO annotations used for the other GO annotations to generate the 40 genes used in the final positive gene set for glutathione metabolic process term. These annotations were retained due to the small number of genes annotated by experimental evidence for the GO term, thus creating a larger positive gene set for model training. bioDBnet [49] was used to link gene symbols from AmiGO to Ensembl gene IDs found in the CCLE transcriptomics data.

The negative classification gene set was generated by randomly sampling from genes that were not included in the positive set based on GO annotations. Genes were randomly selected from the unannotated gene set (i.e. not positive genes) without replacement until an equivalent number of genes to the positive samples were selected. This resulted in balanced positive and negative genes (samples) for model training and testing. However, training a model through this process is biased by the specific set of randomly selected negative class genes in the training set. To ensure robustness of the models, 100 bootstrap iterations of random sampling were performed to create different sets of samples for model training. Final predictions and evaluations are average results over training and testing iterations on the 100 different data sets.

#### Model development and feature selection process

To determine the best methodology for our task, several common ML algorithms and feature sets were tested. Random Forest (RF), Decision Tree (DT), Naïve Bayes (NB), and Support Vector Machine (SVM) classifiers were trained and tested for our three independent classification tasks of mitochondrial, transmembrane transporter activity, and GSH metabolic process GO terms (see Supp. Tables 1 & 2 for algorithm parameters). Models were tested with several sets of features that combine experimental features from CCLE data with knowledge-based features, or feature sets containing only experimental features. Experimental feature sets include the first 5, 14, 30, or 50 CCLE transcriptomics PCs which are selected to capture 93%, 95%, 97%, or 98% of the variance, respectively (Supp. Fig. 2a). For a set of PCs to be used as features, component values are used directly as features. For classifying the GSH metabolic process GO term, Spearman correlations values between gene transcriptomics and GSH or GSSG metabolomics were included as features as well. Features from existing biological knowledge were included through MitoCarta scores for mitochondrial localization in the mitochondrial classification task, and TrSSP prediction scores for membrane transport proteins in the transporter classification task.

For comparisons of feature sets in models, either metabolomics or knowledge features were replaced with additional PC features to create the transcriptomics-only models with an equivalent number of features (i.e., a mitochondrial classifier with the first 5 PCs and two MitoCarta score vectors as features is comparable to a transcriptomics-only classifier using the first 7 PCs as features). This gave transcriptomics-only models with 7, 16, 32, and 52 PC features.

#### Model evaluation

Model performance was analyzed from predicted class probabilities using common evaluation metrics including receiver operating characteristic (ROC) and precision-recall curves (PRC). These methods provide a balanced approach to evaluating classifier performance, which considers the effects of true positives (TPs), true negative (TNs), false positives (FPs), and false negatives (FNs). Models were evaluated and selected based on values for the areas under ROC (AUROC) and PRC (AUPRC), precision, recall, accuracy, F1 score, and Matthew’s correlation coefficient (MCC) [50, 51]. These evaluation metrics were calculated across all test-set predictions from five-fold cross-validations of randomly sampled training/test sets. Reported metrics are calculated as average values across 100 iterations of training/testing using the different gene sets.

#### GEPIA gene expression analysis

For evaluation of gene expression in datasets other than the CCLE data used in classifier models, paired TCGA [45] and GTEx [52] transcriptomics data were obtained via GEPIA2 [53]. This database includes 9736 tumor samples from TCGA and 8587 normal samples from GTEx. Data were obtained from the GEPIA2 webpage (http://gepia2.cancer-pku.cn/).

#### Protein structural analysis

##### SLC25 structures

Protein structures are obtained from the AlphaFold [19] structure database (alphafold.ebi.ac.uk) for use in structural analyses. Additionally, homology models from SWISS-MODEL [54] for both SLC25A39 and SLC25A40 are included in our analyses due to different conformational states of AlphaFold models for these proteins relative to the other SLC25 structure models. Homology based structures are modeled on the crystal structure of the bovine ADP/ATP carrier (PDB:OKC1) [55] and cryo-electron microscopy determined structure of human UCP1 (PDB:8HBV) [56] for SLC25A39 and SLC25A40, respectively.

##### Multiple sequence alignment

Protein sequences for relevant SLC25 proteins were obtained from Uniprot, and multiple sequence alignment (MSA) was performed via the EMBL Clustal Omeg6 [57] web server (https://www.ebi.ac.uk/jdispatcher/msa/clustalo) using default parameters. MSA result visualization and pairwise alignment were determined using Jalview (version 2.11.0) [58, 59].

##### Protein structure alignments

Comparisons of 3D protein structures were conducted using sequence-independent alignment through the TM-align algorithm [60] and quantified by the corresponding TM-scores, which quantifies similarities through similarities in protein structure topology. This method performs comparably to other common alignment methods and does not consider sequence similarity for alignment, which could skew the alignments due to conserved sequences within the SLC25 family. Alignments were implemented using the TMalign module for PyMOL [61] and predicted mGSH transporter structures were compared to known mGSH transporters (SLC25A39, A40).

The CAVER PyMOL plugin version 3.0.3 [62] was used to identify tunnel residues for each SLC25 structure. Tunnels were identified by initializing the tunneling at the base or innermost point of the transporters tunnel with maximum starting point distance and desired radius of 3Å for starting point optimization. From this base, the tunneling algorithm probed outward to generate an interior tunnel channel. CAVER default parameters were specified for the tunneling algorithm, using 12 approximating balls with minimum probe radius of 1.5Å, shell depth of 20Å, shell radius of 7Å (or 10Å for structures where a smaller radius fails to fill the tunnel space), and a clustering threshold of 3.5. Default parameters were used for computation memory and speed, and were adjusted as described, if necessary, for finding transporter tunnels. Relevant tunnels were identified from the top-ranked tunnels identified by CAVER as those tunnels which traversed and filled the length of the proteins’ tunnel without escaping to the protein surface through a side gap or by “overflowing” the interior tunnel.

In the same fashion as the full structure alignments, analysis of the tunnel regions for the SLC25 family were performed by aligning the tunnel-interacting residues identified from CAVER using TM-align.

##### GSH and GSSG docking simulations

Binding of GSH and GSSG to SLC25 protein structures were simulated in PyRx [63] (version 0.80). Metabolite structures were downloaded from the human metabolome database (HMDB) [64, 65]. For each protein structure, tunnel regions, as identified by CAVER, were estimated manually as boundaries for docking simulations. Exhaustiveness values of 8 were used for each docking simulation, reported binding affinities and displayed poses are the top-ranked position returned by PyRx.

## Results

### Classifier models for mGSH transporters in cancer

**Fig. 1:**
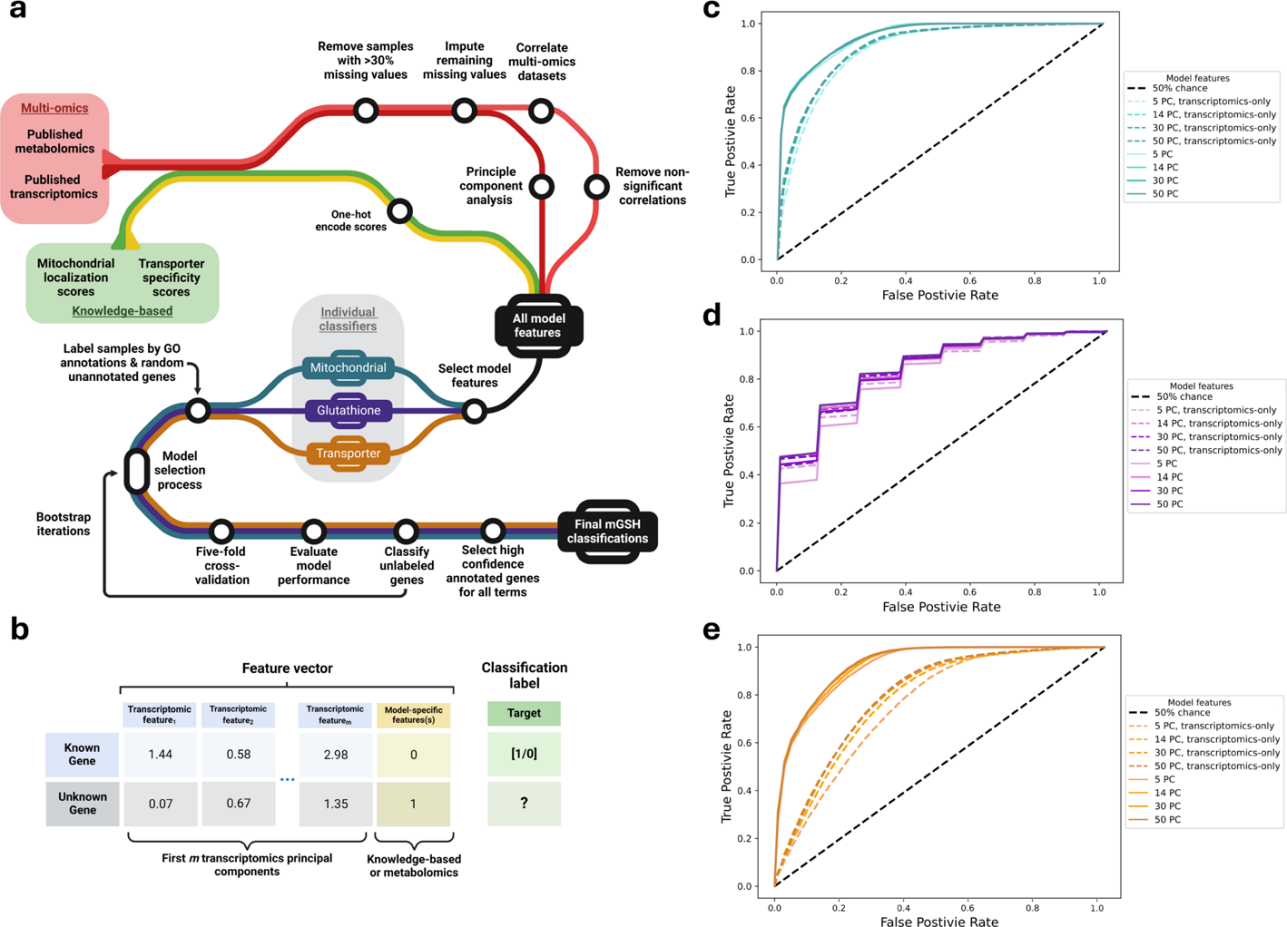
Overview of mGSH transporter classifier model design and evaluation. **a** Schematic of the workflow for ML model development (inspired by designs by Fellows Yates et al. [66]). In this diagram, lines represent the flow of information from the initially collected raw data through several transformation steps to the processed model features, and then through the ML training, evaluation, and selection steps to obtain the final classification of our unknown genes. **b** Example feature vectors assigned to genes in this framework, where transcriptomic features are PCs and model-specific features are MitoCarta scores, for example. Genes in train and test sets are assigned a binary classification based on GO term annotation, novel annotation classification can be applied to unknown genes. ROC curves are presented for mitochondrial (**c**), transporter (**d**) and glutathione (**e**) classifiers with various feature sets. Models which incorporate transcriptomics and other feature data (“hybrid”) are solid while transcriptomics-only models are dashed. Differently shaded curves represent models with different numbers of PC features obtained from transcriptomics data (5–50 PCs for hybrid, comparable transcriptomics-only models have 7–52 PCs)

To identify top mGSH transporter candidates, three independent classifier models were developed to classify genes based on our desired candidate characteristics: mitochondrial localization, association with GSH metabolism, and transmembrane transport function (see Fig. 1a for overview and Methods for details). Genes classified by all three models to possess each characteristic are considered candidate mGSH transporters. Each model uses features from CCLE transcriptomics data along with one or more features from other sources; GSH and GSSG metabolomics correlations for GSH metabolic process classification, MitoCarta scores for mitochondrial localization classification, and TrSSP scores for transmembrane transporter activity classification (Fig. 1b).

Our classifiers performed well in the tasks of mitochondrial, GSH, and transporter annotation classification across our evaluation metrics, as shown in Table 1. Across all models and feature selections, mean five-fold cross-validations shows AUROC is 0.820, and RF classifiers being the best performer with mean AUROC values of 0.900. Classifier performance varied across our three classifier types, with mitochondrial classifiers performing the best and glutathione classifiers performing the worst, on average, which may be attributed to two factors. First, the set of annotated genes related to GSH metabolism is much smaller than the gene sets for the other classifiers (~ 40 genes vs. ~ 700 genes), thus providing a smaller training set for these models. Additionally, the GSH term classifiers rely solely on CCLE multi-omics data unlike the other two classifiers which also include data from existing knowledge-sources.

**Table 1:** Average performance of classifiers in mGSH transporter classification

Algorithm	Metric			
	Model	AUROC	AUPRC	MCC
Decision tree	Hybrid	0.7663	0.8270	0.5371
	Transcriptomics-only	0.6896	0.7692	0.3840
Naïve-Bayes	Hybrid	0.8553	0.8422	0.3517*
	Transcriptomics-only	0.7433	0.7334	0.2028*
Random forest	Hybrid	**0.9003**	**0.8941**	**0.6349**
	Transcriptomics-only	**0.8380**	**0.8156**	**0.5437**
Support vector machine	Hybrid	0.7614	0.7558	0.3487
	Transcriptomics-only	0.7130	0.7250	0.3125

Scores are mean values across each classifier type using transcriptomics features combined with other feature sources (“hybrid”), or transcriptomics features alone (“transcriptomics-only”). Individual scores are calculated as averages across bootstrap iterations. The highest scoring model for each evaluation metric is bolded

^*^Low MCC values relative to other evaluation metrics due to low positive class accuracy

Along with testing several ML algorithms in our classifiers, multiple feature sets were explored to determine the best selection for our task. Specifically, variations of the number of top PCs as linear combinations of transcriptomic features (described in Methods) from 5 to 50, were tested. Increasing the number of these features in our models did not notably improve performance by our metrics (Supp. Fig. 1b). Based on these results, a small number of the first several PCs are sufficient for effective classification. However, specific gene classification probabilities are variable across models with different numbers of PC features used. Since performance is similar across these models, rather than selecting a specific model, we used mean gene classification probabilities with standard error (SE) calculated from models with different numbers of PC features (5, 14, 30, 50) for final classification results.

### Comparison of hybrid models and transcriptomics-based models

Consistently across all tested models, classifiers that incorporate both experimental data from CCLE transcriptomics data and data from other knowledge bases as features outperform comparable models where knowledge-based features are replaced with additional experimental data features (Fig. 1c, d., Table 1, Supp. Fig. 1c). Models contain a total of 7, 16, 32, or 52 features. Although no knowledge component exists for the GSH term classifiers, transcriptomics-based models were compared to models which incorporate GSH and GSSG metabolomics data from the CCLE along with the CCLE transcriptomics data for features. Interestingly, the additional data source does not seem to similarly improve performance in the GSH term models, with performance of transcriptomics-only GSH term classifiers comparable to classifiers using both transcriptomic and metabolomic features (Fig. 1e).

Supporting these findings, classifier feature importance defined as the mean impurity decrease across random forest trees illustrates that while MitoCarta and TrSSP scores are amongst the most important features, metabolomic correlations are the least important for the GSH classifier (Supp. Fig. 1d, e). Interestingly, certain low explained variance (high rank number) transcriptomics features appear to be relatively important -indicating function-specific variations captured by those components.

### Comparison of hybrid models to knowledge-based models

**Fig. 2:**
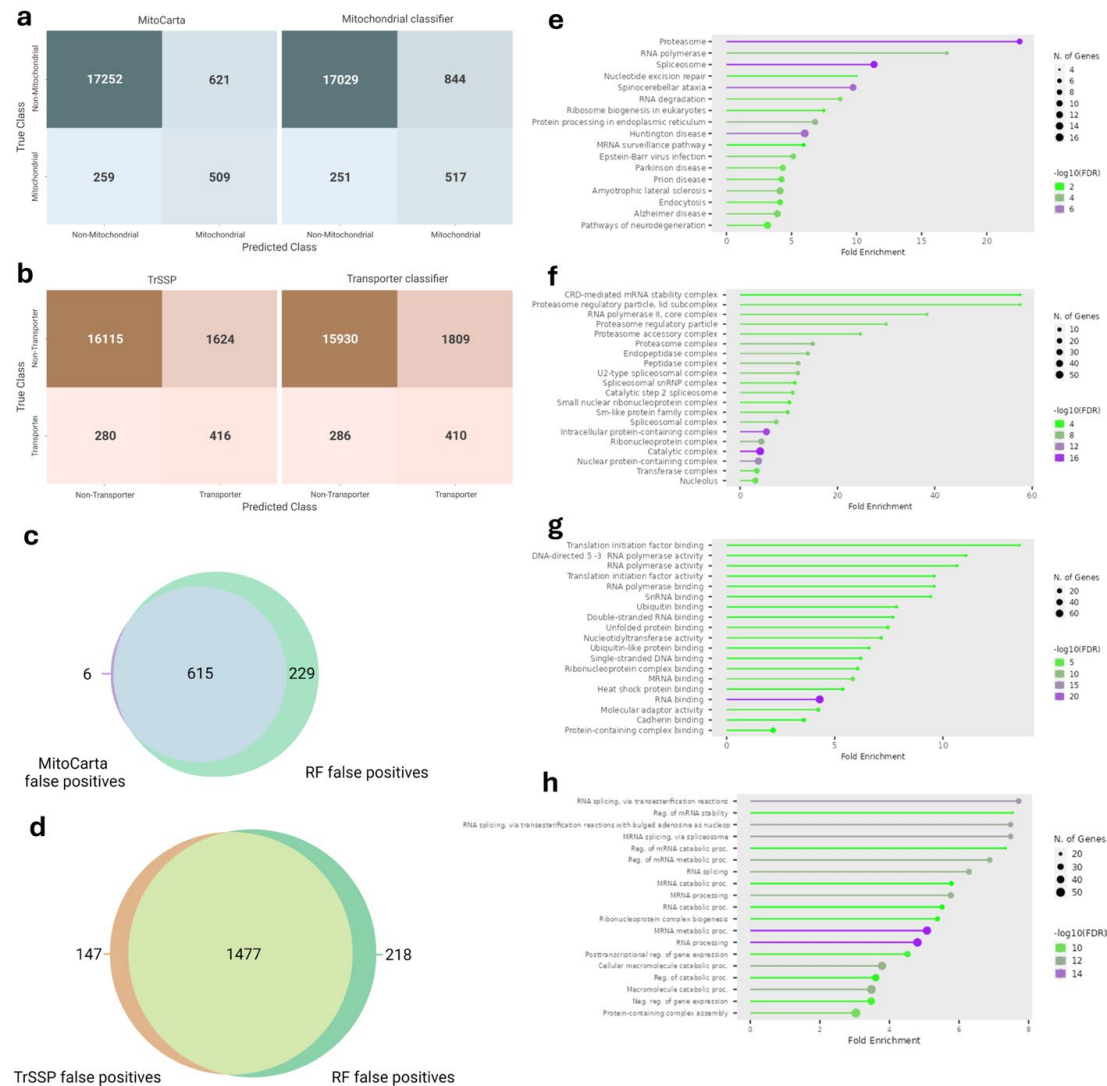
Confusion matrices of existing mitochondrial (MitoCarta) and transporter (TrSSP) models (left) and mean of proposed RF classifiers (right). **a** MitoCarta annotations compared to mean mitochondrial RF classifier annotations. **b** TrSSP compared to mean transporter RF classifier annotations. All unannotated genes are included as negative samples in matrices. Matrices are colored based on the number of samples contained within each quadrant. A classification threshold of 0.75 was used for the mitochondrial and transporter classifiers as it provided the best balance of maximizing true positives and minimizing false positives. **c** Venn diagram of false positive mitochondrial genes predicted by MitoCarta 3.0 and mitochondrial RF classifiers. **d** Venn diagram of false positive transporter genes predicted by TrSSP and transporter RF classifiers. **e**–**h** GO enrichment analysis of genes predicted to localize to mitochondria by RF classifiers with no existing evidence in localization databases. Enrichment plots of false positive mitochondrial genes for KEGG pathways (**e**) cellular component (**f**), molecular function (**g**), and biological process (**h**) GO terms

Like the previous analysis, we sought to evaluate the effects of adding transcriptomics information to the existing predictive models from the literature. Mean classifications from the hybrid RF mitochondrial and transporter classifiers using 5, 14, 30 and 50 PC features were compared to those of MitoCarta and TrSSP. The combined transcriptomic and MitoCarta/TrSSP classifiers appear to only show slight differences to the existing models (Fig. 2a, b). The mitochondrial classifier identifies slightly more mitochondrial genes than MitoCarta alone, however, there are more false positives (FPs) in this classifier (at a classification threshold of 0.75).

### Novel mitochondrial and transporter annotations from hybrid models

While the number of true positives and false negatives are similar, the RF classifiers predict more false positives and less true negatives compared to the results of MitoCarta and TrSSP. To further explore differences between the models, and the potential for identifying novel functions, mitochondrial FP genes were compared to other sources of mitochondrial genes beyond the high confidence GO annotations used for classifier training. Low confidence GO annotations that were originally removed from the training set, Human Protein Atlas subcellular localization annotations from histology images [67], Swiss-Prot annotations [68], and integrated mitochondrial protein index (IMPI) annotations based on MitoMiner [69] were used for other sources of localization evidence. Of the 844 FP genes predicted by the mitochondrial RF classifier, 615 were predicted by both MitoCarta and RF, and 229 were predicted by RF only (Fig. 2c). 206 of these RF FP genes have no evidence in the other datasets, while the remaining have evidence in at least one source (Supp. Data). Enrichment analysis revealed that the 206 unannotated FPs without existing evidence are significantly enriched in KEGG pathways (Fig. 2e) and GO terms (Fig. 2f–h) that are associated with degradation pathways, such as the proteasome, RNA stability, degradation and processing pathways, and are furthermore highly associated with one another based on high confidence evidence through STRING [70] (Supp. Fig. 3a). The proteasome system is closely linked to mitochondrial remodeling in several physiological and disease states, including cancer [71, 72].

Comparisons of FPs in TrSSP and our transporter RF classifier find less overlap between the two (Fig. 2d). 147 FPs are exclusive to TrSSP, 218 are exclusive to the RF classifier, and 1477 are common to both models. In the same fashion as mitochondrial genes, other evidence sources beyond the high confidence GO term annotations were compared to the FPs predicted by transporter models. Low confidence AMiGO annotations, Transporter Classifier Data Base annotations [73], and the SLCAtlas [74] were used as alternate evidence sources. Swiss-Prot annotations were excluded as these were used in TrSSP model training. For both TrSSP and our RF classifier, only a small number of FP genes have evidence from other sources (2 for TrSSP, and 8 for the RF classifier, Supp. Data). Unlike the mitochondrial FPs by our RF classifier, transporter FP genes are not extensively enriched in specific GO terms (Supp. Fig. 3b–d), and no enrichment of KEGG pathways is observed. The most apparent enrichment is in biological process terms related to cell–cell interactions, such as cell adhesion, cell junctions, and synapses (Supp. Fig. 3d).

While the majority of predicted functional annotations are similar between knowledge-based models (MitoCarta or TrSSP), RF classifiers combining these features with transcriptomics features diverge mainly in terms of novel positive predicted annotations (false positives). Exploring these genes reveal notable enrichment of certain functional terms relevant to cancers.

### mGSH transporter candidates

Considering the top SLC25 transporters by mean GSH probabilities, the classifiers identify several potential mGSH transporters. The top 10 SLC25 family members by GSH probability have mean probabilities across RF classifiers greater than 0.73 and includes the known mGSH transporter SLC25A39 (Table 2). Surprisingly, SLC25A40, the homolog of SLC25A39, has a very low GSH probability by the RF classifier models and is not predicted to be related to GSH. Low expression of SLC25A40 relative to SLC25A39 may explain the lack-of GSH function annotation (Supp. Fig. 4). Amongst the top 10 candidates are several well characterized proteins such as SLC25A1, SLC25A10, and SLC25A11 as well as some orphan members like SLC25A43 and SLC25A50. To understand the possible roles of these members in GSH transport, existing evidence from the literature is described in the Discussion section.

**Table 2:** Top 10 SLC25 proteins by mean GSH RF classifier probability

Rank	Gene Symbol	GSH probability
1	SLC25A1	0.7891 (0.0173)
2	SLC25A10	0.7800 (0.0256)
3	SLC25A13	0.7737 (0.0156)
**4**	**SLC25A39**	**0.7716 (0.0230)**
5	SLC25A50	0.7620 (0.0222)
6	SLC25A43	0.7603 (0.0318)
7	SLC25A24	0.7577 (0.0345)
8	SLC25A37	0.7494 (0.0260)
9	SLC25A3	0.7410 (0.0489)
10	SLC25A11	0.7322 (0.0074)
**37**	**SLC25A40**	**0.4702 (0.0152)**

GSH probabilities are mean probabilities for GSH metabolism GO term classification with standard error for RF classifiers over different feature sets (5–50 PC features). The known GSH transporters SLC25A39 and SLC25A40 are in bold

### Non-SLC25 mGSH-related proteins

Alongside the SLC25 family, the remaining genes in our dataset were considered for roles in mGSH transport. Top candidates were identified by their mean GSH probability across RF classifiers. Additionally, the mean mitochondrial and transporter classifier scores were considered. To identify high confidence candidates and minimize potential false positives, only genes with probabilities greater than 0.80 across all classifications are selected (i.e., genes already annotated with one of our GO terms are said to have probabilities of 1). This thresholding removes 99.95% of the available 49,309 genes in the datasets, leaving only 27 genes that meet these strict criteria (Supp. Table 3). Of these, 4 genes are already annotated with the glutathione metabolic process GO term, 13 are annotated with the mitochondrion GO term, and 5 with the transmembrane transporter activity term.

A number of these genes have previously been associated with GSH, in particular we highlight Pyruvate Carboxylase (PC) [75], Mitochondrial calcium uniporter (MCU) [76, 77], ATP-binding cassette family B6 (ABCB6) [78], and neudesin neurotrophic factor (NENF) [79]. We provide a review of GSH associations for these in the Discussion section.

### Transporter structural comparisons

To further examine the SLC25 proteins most probably relevant to GSH transport, we next conducted structural analyses. Experimental determinations of protein structures for the SLC25 family are currently lacking, with the only human SLC25 structure determined for UCP1 by cryo-electron microscopy [56]. Beyond this, structures for orthologs in model species have been determined through X-ray crystallography for the bovine [55] and *Thermothelomyces thermophila* [80] ADP/ATP carriers (SLC25A4), and by NMR molecular fragment searching for the yeast UCP2 (SLC25A8) [81]. Predicted protein structures by AlphaFold and homology modeling must be used for comparison of family members with unknown structures. Particularly, we were interested in structural comparisons between our candidates with the GSH transporters SLC25A39, and SLC25A40 to reveal any similarities in structure and potential function. AlphaFold structures for all relevant SLC25 proteins were obtained in the conformational state open to the cytoplasm and intermembrane space (C-state) except for SLC25A39 and SLC25A40 whose predicted structures are in the M-state, open to the mitochondrial matrix. Thus, homology models for these two transporters, which are in the C-state, are also used in the analysis for comparison. Pairwise structural alignments were determined using TM-align (see Methods) for full 3D structures (“global” alignments) or tunnel-interacting residues within structures (“local” alignments). The residues for local alignments were identified via CAVER [62] (Supp. Fig. 5a). Rather than considering the entire transporter structure, of more relevance to transporter function and specificity is the interior tunnel of the protein surface, along which substrates interact and move across the IMM [80, 82]. By aligning only potentially substrate binding residues we get a more relevant analysis of the SLC25 family with respect to substrate binding as these structures are expected to be more variable and better indicators of shared transport activities.

**Fig. 3:**
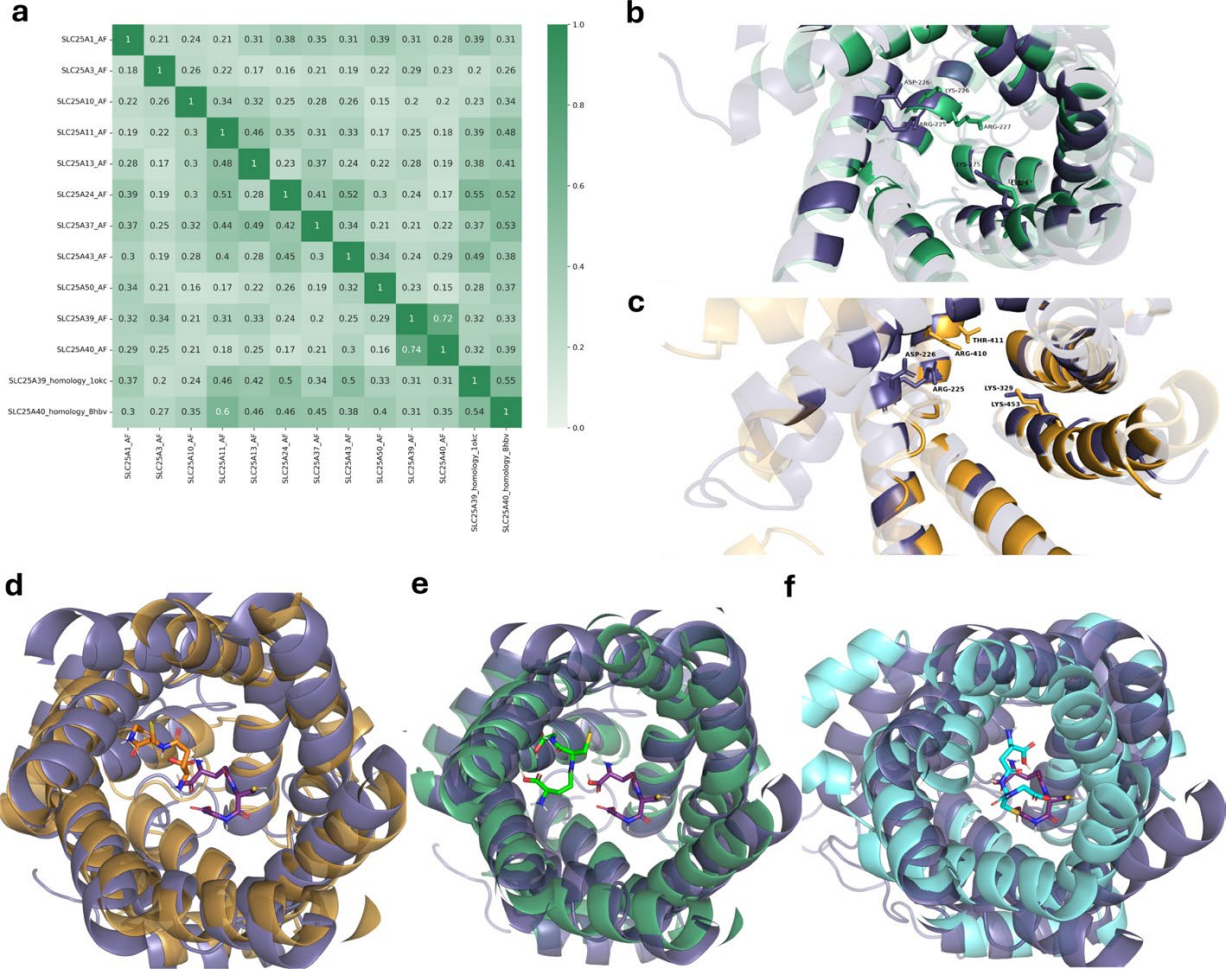
Structural alignment by TM-align of CAVER-identified transporter tunnels in SLC25 family members predicted to be associated with glutathione metabolic processes. **a** Heatmap of TM-scores for pairwise alignments of tunnel-interacting regions identified by CAVER for candidate SLC25 members. Values are TM-scores normalized to the sequence length of the column protein. **b** & **c** Visualization of pairwise transporter tunnel alignments for SLC25A39 (dark purple) and most similar SLC25 candidates by TM-score, SLC25A43 (green) and SLC25A24 (orange). Indicated residues are those identified as relevant for GSH transport by SLC25A39 and corresponding residues in SLC25A43 and SLC25A24. Protein tunnels visualized from the intermembrane space with tunnel-interacting residues identified by CAVER highlighted and non-tunnel residues faded. **d–f** In silico binding of GSH by known and predicted mGSH transporters. GSH-bound SLC25A39 (purple) is aligned to GSH-bound structures of SLC25A43 (orange), SLC25A24 (green), and SLC25A10 (blue). GSH structures are coloured according to their bound protein structure

Pairwise global alignments showed high similarities (TM-scores close to 1) across the transporter family (Supp. Data). This is expected due to common structural domains across the family, namely the six transmembrane helices and evident in regions of high conservation in multiple sequence alignment (MSA) (Supp. Fig. 6) computed for all proteins used in structural and docking experiments.TM-scores for local alignments of tunnel residues are much lower relative to the global alignment scores (mean scores excluding self-alignments 0.376 vs. 0.701) (Supp. Fig. 5b) and more variable (coefficient of variation of 0.350 vs. 0.172 for global alignments), indicating the diversity of transport tunnels and functions of the SLC25 proteins. Figure 3a details the TM-scores for local pairwise alignments between top mGSH candidate SLC25s as well as SLC25A39 and SLC25A40.

Amongst these results, low alignment scores between homology and AlphaFold models for SLC25A39 and SLC25A40 indicate the dramatic changes in conformational state and thus comparisons to the C-state (i.e., homology models) are investigated. Relatively high scores between SLC25A24, SLC25A43, and SLC25A39 indicate similarities in transporter tunnels and possible overlapping transport functions of the proteins. Shi et al. [15] identify R225, D226, and K329 as important residues for GSH transport in SLC25A39. Based on local structural alignments of tunnel regions with SLC25A43 and SLC25A24 (Fig. 3b, c), the position of the K329 residue is conserved by K275 in SLC25A43 and by K453 in SLC25A24. However, the R225, D226 motif of SLC25A39 appears less conserved in the aligned structures. In the SLC25A43 alignment, residues G173 and A174 are in closest proximity to the A39 binding residues, and these non-polar residues are not identified by CAVER as tunnel-interacting residues. Interestingly, the sidechains of SLC25A43 residues K226 and R227 appear near R225 and D226 sidechains in SLC25A39, where the positively charged K226 replaces the negative charge of D226 in SLC25A39. Like SLC25A43, the closest aligned residues of SLC25A24 to the GSH binding residues of SLC25A39 are the non-polar G353 and I354, which do not interact with the transporter tunnel. Where K226 and R227 are found in SLC25A43, instead there is R410 and T411 in SLC25A24.

### GSH and GSSG binding in mGSH transporter candidates

Docking experiments were conducted on transporter proteins using AlphaFold and homology structure models to evaluate GSH and GSSG binding by predicted mGSH transporters. We observe similar binding affinities across protein structures (Table 3), ranging − 6.8– − 5.3 kcal/mol for GSH and − 8.0– − 5.6 kcal/mol for GSSG. Interestingly, SLC25A10 shows the strongest affinity (i.e., more negative) for both GSH and GSSG. SLC25A40 shows a distinct preference for GSSG (− 7.0 kcal/mol) rather than GSH (− 5.6 kcal/mol), it has the weakest affinity for GSH of the structures docked here. SLC25A39 and the predicted transporters, SLC25A24, and SLC25A43 have relatively weak binding with slight preference for GSSG. Binding poses for GSH in SLC25A10, SLC25A24, and SLC25A43 structures are similar to SLC25A39 GSH binding (Fig. 3d–f), with SLC25A24 the most distant.

**Table 3:** Top binding affinities (kcal/mol) for GSH and GSSG in docking experiments for predicted mGSH transporter structure models

Protein structure model	GSH binding affinity (kcal/mol)	GSSG binding affinity (kcal/mol)
SLC25A1_AF	− 5.6	− 5.5
SLC25A10_AF	**− 6.8**	**− 8.0**
SLC25A11_AF	− 5.6	− 6.9
SLC25A13_AF	− 6.2	− 5.9
SLC25A24_AF	− 5.9	− 6.7
SLC25A3_AF	− 6.1	− 7.1
SLC25A37_AF	− 6.0	− 6.8
SLC25A43_AF	− 5.6	− 6.3
SLC25A50_AF	− 6.2	− 6.2
SLC25A39_AF	− 5.7	− 5.8
SLC25A40_AF	− 5.8	− 6.3
SLC25A39_homology_1okc	− 5.9	− 6.4
SLC25A40_homology_8hbv	− 5.6	− 7.0

“_AF” and “_HOMOLOGY_” suffixes indicate the structure is predicted by AlphaFold2 or homology modeling, respectively. The PDB ID for the homology modeling structural template is also included. Strongest binding affinities (most negative) for each ligand are bolded

### Comparison to DeepGOPlus

**Fig. 4:**
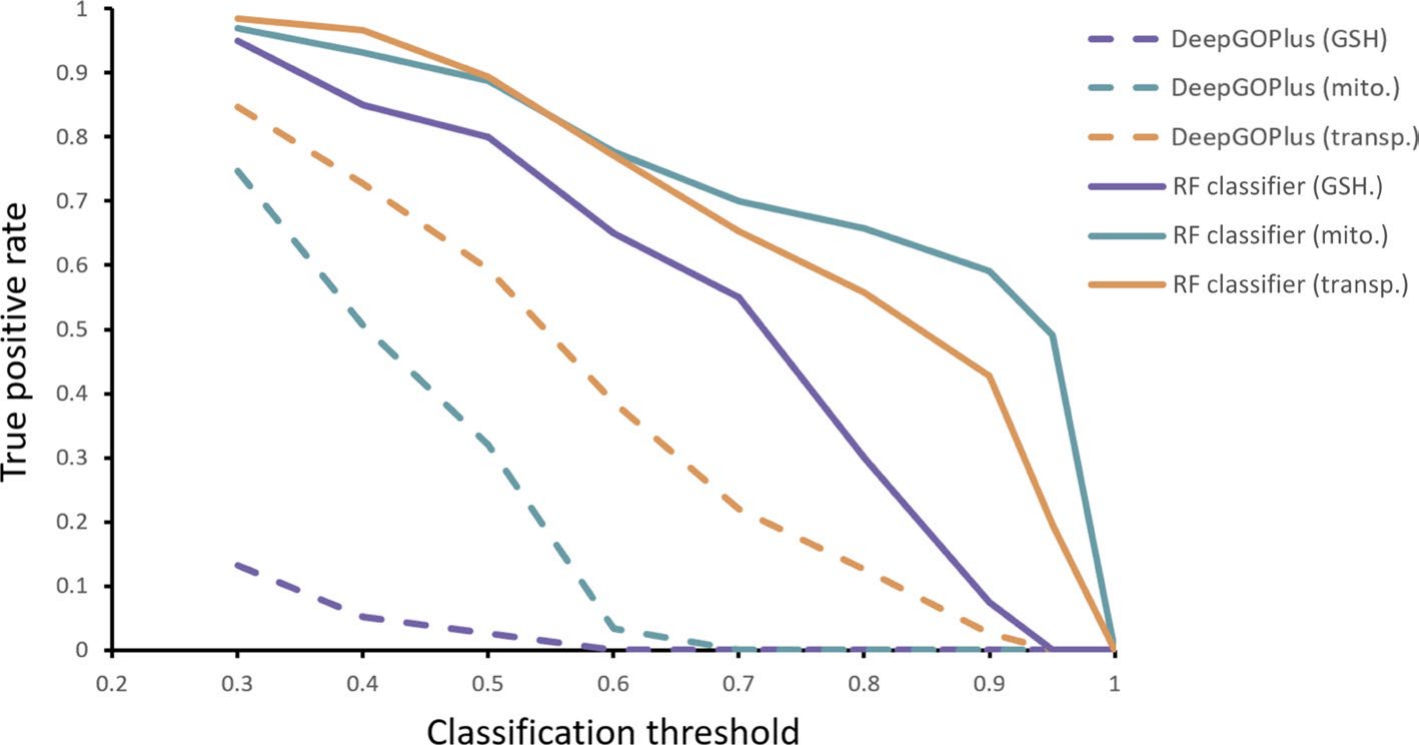
Evaluation of gene ontology annotation predictions by DeepGOPlus (dashed) and RF classifier models (solid). Sensitivity, or TPR, are calculated at various classification thresholds for GSH metabolism, mitochondrial, and transporter GO term annotation tasks

For a comparison to the models developed here, genes from our GO terms of interest were annotated by DeepGOPlus. DeepGOPlus [22] is a general function annotation deep learning model based on protein amino acid sequences. The model is constructed as a convolutional neural network (CNN) in which protein sequences are taken as inputs and the predicted GO term annotation and probabilities are returned. Gene ontology terms with predicted annotation probabilities were obtained through the DeepGOWeb API [83] (Version 1.0.13) using FASTA sequences obtained from canonical Ensembl transcript IDs for the genes under each ontology term. All 39 high confidence glutathione metabolic process annotations, and 150 randomly selected genes from each other ontology term were annotated using DeepGOWeb. The true positive rates (TPRs) or sensitivity measures of annotations were determined for model comparison (Fig. 4, Supp. Table 4).

In all comparisons across classification thresholds and annotation tasks, DeepGOPlus performs worse than the proposed hybrid RF classifier (Supp. Data). This is most evident for glutathione metabolism GO term classification, where DeepGOPlus achieves an average sensitivity of 0.070 over classification thresholds of 0.3–0.5 compared to 0.867 for the RF classifier over the same range. This range was chosen for comparison because DeepGOPlus rarely assigns probabilities > 0.5 for a single GO term due to its multi-classification architecture, favouring the proposed hybrid model in any comparisons using the full range of classification thresholds (see Supp. Data). For mitochondrial localization and transporter activity annotations, DeepGOPlus reaches mean TPRs of 0.525 and 0.722, respectively, though the hybrid RF classifier still outperforms with mean values of 0.930 and 0.948, respectively.

DeepGOPlus is trained to classify all GO terms with at least 50 annotations, as noted, in this multi-classification approach the probabilities assigned for any specific GO term annotation may be inaccurate. This limitation is most notable in the classification of the GSH metabolic process term, which is underrepresented in the training of DeepGOPlus due to its small size.

### Validation of metabolism GO term annotation framework

In comparison to GSH term annotation classifiers, several other classifier models were trained and evaluate the performance on GO term classification for other metabolites. Like the GSH term classifiers, RF models with 5–50 CCLE transcriptomic PCs and corresponding metabolomic correlations as features were trained for the annotation of genes related to 2-oxoglutarate, glutamate, and carnitine. Unlike GSH which has a reduced and oxidized form, these metabolites have only one relevant form, thus one correlation feature is included for each of these metabolite classifiers. Following the procedure for the GSH classification task, positively classified genes were identified from GO terms for these metabolites to generate training and test gene sets for classifiers. 2-oxoglutarate, glutamate, and carnitine were selected for this evaluation because, out of metabolites found in the CCLE metabolomics set, they have a relatively larger number of annotated genes, making classifier training possible. Additionally, there is evidence for transport of each by one or more SLC25 proteins, which allows for some measure of classifier validation.

For each of these classifiers, performance was comparable to the GSH term classifier by our evaluation metrics (Supp. Table 5). Like GSH term classifications, SLC25 candidates were ranked by mean metabolite RF classifier probability (from classifiers with 5–50 PC features) and top 5 candidates are reported (Table 4). Across models, top candidates have a minimum probability of 0.76 by their respective classifiers.

**Table 4:** Top 5 SLC25 candidates for non-GSH metabolites by RF classifiers

Rank	Gene symbol	Glutamate probability	Rank	Gene symbol	2-oxoglutarate probability	Rank	Gene symbol	Carnitine probability
1	SLC25A39	0.7998 (0.0409)	1	SLC25A39	0.8408 (0.0166)	1	SLC25A30	0.8204 (0.0361)
2	SLC25A1	0.7889 (0.0357)	2	**SLC25A11**	**0.8304 (0.0228)**	2	SLC25A29	0.8116 (0.0196)
3	SLC25A50	0.7784 (0.0322)	3	SLC25A1	0.8287 (0.0351)	3	SLC25A36	0.7682 (0.0131)
4	SLC25A5	0.7706 (0.0148)	4	SLC25A28	0.8060 (0.0145)	4	SLC25A25	0.7632 (0.0249)
5	SLC25A10	0.7617 (0.0248)	5	SLC25A10	0.8059 (0.0292)	5	SLC25A38	0.7618 (0.0211)
11	**SLC25A13**	**0.7378 (0.0305)**				28	**SLC25A20**	**0.6415 (0.0345)**
23	**SLC25A22**	**0.6847 (0.0304)**						
36	**SLC25A12**	**0.6139 (0.0469)**						
42	**SLC25A18**	**0.4663 (0.0178)**						

Candidates ranked by mean RF classifier probabilities (5–50 PC features) with standard error. Known metabolite transporters are bolded

### Robustness of CCLE transcriptomics principal components

Alternate principal components were generated from CCLE transcriptomics data and compared to the whole dataset, “baseline” components used in classifier models to evaluate the effect of sample selection, size, and biological conditions on the gene expression variances captured by PCA. Principal components generated from whole CCLE transcriptomics explain most variance in the first 10 components (Supp. Fig. 2a) and through random sampling of the data, principal components generated from smaller subsets of the dataset behave similarly (Supp. Fig. 2b), where the first components explain similar variances. Higher ordered components are less similar, in proportion to the number of random samples.

Along with randomly subsetting the dataset, samples were selected according to the 23 cell line-associated tissue types, and principal components generated. Though the number of samples, and thus principal components, for each tissue-type varied, similar patterns of explained variance are observed across tissue-types compared to the baseline components (Supp. Fig. 3a).

We also observe that, like the random sampled components, tissue-type components are highly similar to the baseline components, with tissue-specific variations explained by the higher-ranked components (Supp. Fig. 7). Additionally, there does not appear to be any relationship between similarity of tissue-type components to the baseline and the number of tissue-type samples, indicating that the larger tissue-type samples (for example, lung) do not bias the baseline components.

### GO term annotation with cancer-specific cell line and primary tumor classifiers

To investigate the influence of gene expression on predicted GO annotation by classifier models, several additional RF classifiers were trained using different gene expression datasets. First, tissue-specific CCLE PCs were used as classifier features to develop several tissue-type classifiers. These classifiers follow the framework for mitochondrial and transporter transcriptomics-only models, where classifiers have 7, 16, or 32 transcriptomics PC features, with identical sampling, bootstrap iterations, and training regimes. Due to the lack-of samples in datasets, no 52 PC feature classifiers were used and liver CCLE classifiers contain a maximum of 24 PC features rather than 32. In total, individual mitochondrial and transporter classifiers were trained and tested on three subsets of CCLE samples from skin cutaneous melanoma, pancreatic adenocarcinoma, or liver hepatocellular carcinoma.

Classifier models trained on specific sample subset retain similar, or slightly lower in the case of transporter classification, performance as measured by our evaluation compared to whole CCLE transcriptomics-only models (Supp. Table 6). The major variances in gene expression captured by tissue-specific samples sufficiently classify GO functional terms. Further illustrating this point, despite biological differences between the CCLE subsets we observe that the various classifiers show good agreement in their predicted GO term annotations of unlabeled genes. Pearson correlations for genes with annotation probabilities greater than 0.6 or less than 0.4 were computed for pairs of classifiers for mitochondrial and transporter classification tasks. This finds that predicted annotations are correlated across datasets, with median Pearson correlation values (± standard deviation) of 0.93 ± 0.01, and 0.78 ± 0.02 with pairwise correlation *p*-values < 0.001 for mitochondrial and transporter classifications, respectively.

To further validate this, gene expression values from TCGA primary tumor samples were used to train classifier models. Classifiers were trained to most closely mirror CCLE classifiers, using either all TCGA samples for cancer types found in the CCLE, or the same subsets of skin cutaneous melanoma, pancreatic adenocarcinoma, or liver hepatocellular carcinoma samples. TCGA classifiers used 7, 16, or 32 transcriptomics PC features for annotation mitochondrial or transport GO terms. The whole data TCGA classifiers include 52 PC features, and classifiers trained for GSH GO term annotation.

Classifiers using TCGA transcriptomics features show similar, though consistently lower, performance metrics when compared to their most similar CCLE classifier (Supp. Table 6). Lower explained variance by components of TCGA transcriptomics (Supp. Fig. 9, Supp. Data) relative to CCLE likely contribute to the decreased performance. Median cumulative explained variance by the first 10 PCs across CCLE and TCGA cancer-type datasets are 0.985 ± 0.003 and 0.877 ± 0.047, respectively. Pearson correlations for confident annotations (i.e., greater than 0.6, or less than 0.4) of unlabeled genes show correlations of 0.83 ± 0.01 and 0.71 ± 0.03 (*p*-values < 0.001) between TCGA tumor types for mitochondrial and transporter classifications, respectively. Pairing cell lines and primary tumors from the same cancer type gives median correlations of 0.77 ± 0.03 and 0.29 ± 0.06 (*p*-values < 0.001) for mitochondrial and transporter classifications. Unsurprisingly, differences in extracellular environments, membranes, and thus transporters, between cell lines and in vivo tumor samples contribute to substantially to differences in transporter classifications, but less-so to mitochondrial classifications. Other TCGA-CCLE divergences may result from a combination of overall lower TCGA classifier performance, and low gene expression correlations between paired CCLE-TCGA sample [34, 46].

As a final validation, a GSH GO term classifier was trained using whole TCGA transcriptomics as features, for comparison to mGSH candidates predicted by the hybrid CCLE GSH classifier. Again, the TCGA classifier displays slightly lower performance metrics compared to the CCLE classifier (Supp. Table 6). We compare the top 10 SLC25 proteins by GSH probability predicted by either model (Table 5) and note that despite many differences between the two models, roughly half of the SLC25 family members are common to both. In particular, the most confident top 5 candidates in either and placement of the known mGSH transporters SLC25A39 and SLC25A40 are similar between models—indicating common patterns between datasets and across cancer subtypes which is supported by relative expression levels in TCGA and GTEx (Supp. Fig. 9).

**Table 5:** Top 10 SLC25 proteins by mean GSH RF classifier probability predicted by CCLE and TCGA classifiers

CCLE top SLC25 GSH candidates			TCGA top SLC25 GSH candidates		
Rank	Gene	GSH prob. (std)	Rank	Gene	GSH prob. (std)
1	SLC25A1	0.7891 (0.0173)	1	**SLC25A10**	**0.7092 (0.0969)**
2	**SLC25A10**	**0.7800 (0.0256)**	2	**SLC25A39**	**0.6492 (0.0490)**
3	**SLC25A13**	**0.7737 (0.0156)**	3	**SLC25A50**	**0.6458 (0.0368)**
4	**SLC25A39**	**0.7716 (0.0230)**	4	SLC25A5	0.6333 (0.0775)
5	**SLC25A50**	**0.7620 (0.0222)**	5	SLC25A42	0.6071 (0.0428)
6	SLC25A43	0.7603 (0.0318)	6	SLC25A25	0.5944 (0.0809)
7	SLC25A24	0.7577 (0.0345)	7	**SLC25A11**	**0.5931 (0.0550)**
8	SLC25A37	0.7494 (0.0260)	8	**SLC25A13**	**0.5894 (0.1315)**
9	SLC25A3	0.7410 (0.0489)	9	SLC25A4	0.5743 (0.0925)
10	**SLC25A11**	**0.7322 (0.0074)**	10	SLC25A28	0.5737 (0.0456)
**37**	**SLC25A40**	**0.4702 (0.0152)**	**41**	**SLC25A40**	**0.3231 (0.0373)**

GSH probabilities are mean probabilities with standard deviation for RF classifiers over different feature sets (5–50 PC features for CCLE classifier, 7–52 for TCGA classifier). The known GSH transporters SLC25A39 and SLC25A40 and any SLC25 proteins common to both models are in bold

## Discussion

The importance of mitochondria and GSH in cancers is widely appreciated. Mitochondria have a major role in metabolic shifts, one of the hallmarks of cancer, while mGSH is important in metabolism, ROS processing, post-translational protein modifications and tumor drug resistance. An improved understanding of mitochondrial transport mechanisms in cancer cells, including GSH transport mechanisms, is important for advancing the cancer biology field. To this end we developed a hybrid ML framework that utilizes multi-omics data and existing knowledge for annotating genes with GO terms of interest. We applied this model to the problem of mitochondrial GSH metabolism, specifically focusing on the aspect of transport. We propose several potential candidates that are predicted to be related to, and possibly modulated by, mGSH transport that are identified by this model to guide future experimentation in this area of research.

Random forest classifiers were developed here for the annotation of mGSH transporter characteristics: mitochondrial localization, relation to GSH metabolism, and transporter function. Models combine CCLE transcriptomics and metabolomics data for annotation of genes related to GSH metabolism, and knowledge from existing predictive models for mitochondrial localization and transporter activity annotation. This framework resulted in models with high performance (mean RF AUROC of 0.900) across each classification task. Furthermore, the proposed method is effective even with the minimal number of CCLE multi-omics features that were tested (5 PCs), achieving, at worst, an AUROC of 0.808 for GSH metabolism classification. By the performance metrics used here, the hybrid framework outperforms comparable RF classifiers utilizing only transcriptomics features for classification. Hybrid models were also compared to the existing “knowledge-based” models for mitochondrial genes and transporters, MitoCarta and TrSSP, respectively. The hybrid models show similar performance by confusion matrices, but we observe increased false positive classifications relative to the knowledge-based models. These genes may represent shifts in function in cancer cells compared to normal cells, or genes associated with mitochondria or transporters but which themselves do not possess these functions. Evidence for functionally associated genes is observed in enrichment of proteasome genes for false positive mitochondrial genes, and for cell–cell interaction genes for false positive transporter genes.

For comparison, we attempted similar classifications using DeepGOPlus [22, 83] for annotation of our terms of interest. This general GO annotation model performed worse, as measured by sensitivity in correctly classifying annotated genes, than our framework. This is most evident in GSH metabolic process classification. The mutli-classification GO term task for DeepGOPlus may explain this poor performance, where relatively small set of annotated GSH metabolism term is underrepresented relative to the many other, well-annotated, GO terms during training. Annotating small GO term gene sets is a weakness of these general frameworks and a strength of our proposed approach. Furthermore, as a sequence-based approach, DeepGOPlus cannot be applied to disease contexts as we do with our proposed method, in this case using cancer cell transcriptomics and metabolomics data to focus analysis on a cancer context.

As a further validation of our methodology, several other classifiers using our framework were developed and tested for annotating other GO terms. Identical RF models to the GSH term classifier were developed which instead use genes annotated with GO terms for glutamate, 2-oxoglutarate, and carnitine metabolic process for training. These models show similar performance to the GSH term classifier despite a small number of annotated genes for training, indicating the robustness of this framework for annotating a variety of biological functions. Furthermore, with respect to our interest in transport, these classifiers correctly identify many previously known SLC25 transporters as their most probable candidates.

While whole cancer dataset predictions are explored in detail, to understand the effect of different biological datasets in classifiers, specific cancer types were investigated. Classifiers trained on selections of samples from specific cancer types, skin cutaneous melanoma, pancreatic adenocarcinoma, and liver hepatocellular carcinoma have both comparable performance to whole cancer classifiers in mitochondrial and transporter classification tasks and strong agreement of predicted annotations for unlabeled genes. Furthermore, classifiers using gene expression from TCGA primary tumor samples rather than cancer cell lines show similar performance and strong agreement in mitochondrial gene classifications and divergent transporter classifications.

Considering the SLC25 family of mitochondrial carriers, we find several predicted to be related to GSH metabolic processes. Most notably, SLC25A39, the recently identified GSH transporter, is amongst our top hits (ranked 4th). Furthermore, several SLC25 which are known to be relevant to mGSH metabolism are amongst the top candidates: SLC25A1 [84, 85] (ranked 1st), SLC25A10 [86, 87] (ranked 2nd), SLC25A13 [88] (ranked 3rd), and the iron transporter SLC25A37 [15] (ranked 8th). Surprisingly, in both CCLE and TCGA datasets, the homolog of SLC25A39, SLC25A40, is not predicted to be involved in GSH metabolism, ranking 37th of the 53 SLC25 family proteins. This may be due to the very low expression of SLC25A40 across the CCLE cell lines (Supp. Fig. 4), suggesting that SLC25A40 is quantitatively less relevant to mGSH transport in cancer cells. Similar expression patterns are also observed in TCGA tumor and GTEx normal samples accessed via GEPIA (Supp. Fig. 9). SLC25A40 expression is much lower than SLC25A39 expression, which is increased in many tumor samples compared to the paired normal samples, while SLC25A40 changes are minimal. In agreement of this, classifiers trained on TCGA gene expression data maintain a high SLC25A39 GSH probability, but low SLC25A40. Other potential mitochondrial carriers of interest include SLC25A43 (ranked 6th), SLC25A24 (ranked 7th), and the OMM transporter SLC25A50 (ranked 5th). SLC25A43 is an orphan transporter which has recently been found to affect redox homeostasis [89]. In both TCGA and CCLE GSH metabolism classifiers, SLC25A10 and SLC25A50 are amongst the most probable SLC25 proteins, which may be due to their close associations with energy metabolism [86, 87, 90], of which GSH is crucial.

Structural similarities in the candidates, specifically within the tunnel region of the proteins, can inform potential roles of these transporters in mGSH metabolism. We investigated this through sequence-independent structural alignment of SLC25 transporters using TMalign. We find that, aside from its homolog SLC25A40, SLC25A39 shows the most similarities to our candidates SLC25A43, and SLC25A24. Important GSH binding residues in SLC25A39 are somewhat conserved in our candidates where, specifically, SLC25A43 shows a shift in residue position within the transporter tunnel and a substitution of an aspartate to a lysine residue, and SLC25A24 substitutes a threonine. These findings indicate that despite overall similarities in transporter tunnels between the GSH transporter and our candidates SLC25A43 and SLC25A24, substrate binding and transport mechanism are likely somewhat different, e.g., binding to alternate forms of GSH species such as GSSG or glutathione esters.

Expanding our search beyond the SLC25 family, 27 genes were classified with high probability (> 0.80) for all classification tasks. Of these, some of the most probable candidates are discussed here for their possible roles in mGSH metabolism and transport.

Pyruvate carboxylase (PC) catalyzes the conversion of pyruvate to oxaloacetate in mitochondria. PC links glucose to GSH synthesis and plays roles in the control of redox and oxidative stress [75]. Recently, pyruvate metabolism has been associated with GSH metabolism and ferroptosis in lung cancer cells through the plasma membrane cysteine transporter SLC7A11 [91] (ranked 3rd by GSH metabolic process GO term annotation probability in RF classifiers). PC is reported to specifically localize to the mitochondrial matrix with little evidence supporting it being membrane-bound; thus, it may be that PC interacts with the IMM-bound transporters to facilitate and modulate mGSH transport.

Mitochondrial calcium uniporter (MCU) is also a top hit in our models and a well-known mitochondrial transporter. Associated with its role in Ca^2+^ homeostasis are roles in iron and redox homeostasis, and cell death pathways [76]. With respect to GSH, MCU is regulated via s-glutathionylation [76] and downregulation is associated with enriched GSH metabolism in melanoma cells [77]. It is possible that MCU plays a greater role in mitochondrial iron and GSH metabolism and transport in the context of cancer metabolism than has been reported thus far.

ATP-binding cassette family B6 (ABCB6) is a transporter protein with evidence for both plasma membrane and mitochondrial localization [92]. This protein transports the heme precursor porphyrin [93], however the mechanism of transport has been shown to be GSH-dependent, with significantly increased activity in the presence of GSH [78]. These suggest GSH-mediated transport of substrates by ABCB6, but it is unclear if GSH merely modulates transport, or if it is transported as well, likely through GSH-conjugated species. Similarly, neudesin neurotrophic factor (NENF) is a mitochondrial localized protein primarily involved in the differentiation/development of neuronal cells [94]. NENF activity is modulated by the binding of heme [79], which presents a connection to GSH that, to the best of our knowledge, has not yet been explored. Some evidence suggests NENF is membrane-associated [94].

The results of our models find several genes that may play roles in mGSH metabolism and transport, and thus could guide future experimental analyses. Our findings are limited by our relatively small training dataset, in which more data would improve model performance and confidence of predictions. Furthermore, our models are generalized across cancer cell data from a range of tissue and cancer types. Future work, leveraging larger datasets, or datasets of specific cancer types would provide promising avenues for uncovering interactions of high relevance to the specific diseases. Another limitation of this work is the use of gene ontology terms for identifying relevant genes. Specific and manually curated databases for these functions, similar to MitoCarta, likely provide a more accurate set of relevant genes; however, these databases are limited. Using a database like gene ontology provides a resource for the annotation of many functions, processes, and cell localizations as demonstrated here, which cannot be replicated by specific databases.

Finally, the classifications from the individual models provide novel candidates for areas beyond the scope in this work. For example, the classifiers for other metabolites here were used for validation of the glutathione classifier, but predicted relevant genes for those metabolites were not investigated. Nevertheless, we anticipate that our findings here can be instrumental in the identification of potentially novel proteins involved in mitochondrial glutathione metabolism and transport in cancer cells. The results here and classifier code provide a tool to accelerate knowledge discovery and identify potential target genes for other biological functions and contexts.

## Supplementary Information

Additional file 1.

Additional file 2.

## Data Availability

All data and code used and generated in this work are publicly available at https://github.com/lkenn012/mGSH_cancerClassifiers [95].

## Abbreviations

GSH: Glutathione
mGSH: Mitochondrial glutathione
ML: Machine learning
DL: Deep learning
ROS: Reactive oxygen species
OXPHOS: Oxidative phosphorylation
KEGG: Kyoto encyclopedia of genes and genomes
CCLE: Cancer cell line encyclopedia
TCGA: The cancer genome atlas
GO: Gene ontology
TrSSP: The transporter substrate specificity prediction server
GSSG: Glutathione disulfide
PCA: Principal component analysis
PC: Principal component
RF: Random forest
DT: Decision tree
NB: Naïve Bayes
SVM: Support vector machine
ROC: Receiver operating characteristic
PRC: Precision-recall curve
TP: True positive
TN: True negative
FP: False positive
FN: False negative
AUROC: Area under receiver operating characteristic curve
AUPRC: Area under precision-recall curve
MCC: Matthew’s correlation coefficient
CNN: Convolutional neural network
TPR: True positive rate
