# Supplementary material for: A hybrid machine learning framework for functional annotation of mitochondrial glutathione transport and metabolism proteins in cancers
Source: BMC Bioinformatics. 2025 Feb 11;26:48. doi: 10.1186/s12859-025-06051-1 (PMC11817629; doi:10.1186/s12859-025-06051-1)
Supplement: Supplementary file 2 — Additional file 2. [file 12859_2025_6051_MOESM2_ESM.docx]

**Supplementary file:** “A hybrid machine learning framework for functional annotation of mitochondrial glutathione transport and metabolism proteins in cancers.” Kennedy et al. 2024

| **Algorithm Parameters** | | | | | | | |
| --- | --- | --- | --- | --- | --- | --- | --- |
| **Algorithm** | **Kernel** | **Variance smoothing** | **C (regularization)** | **Gamma** | **Tolerance** | **Shrinking** | **Decision function** |
| Naïve-Bayes | Gaussian | 1e-9 | *NA* | *NA* | *NA* | *NA* | *NA* |
| Support Vector Machine | Gaussian | *NA* | 1.0 | Scale | 0.001 | True | One-vs-rest |

**Supplementary table** **1**. **Hyperparameters for ML algorithms tested for GO term classification.**

| **Algorithm Parameters** | | | | | |
| --- | --- | --- | --- | --- | --- |
| **Algorithm** | **Estimators** | **criterion** | **Splitting strategy** | **Max features** | **Max depth** |
| **Decision Tree** | 1 | Gini | Best nodes, minimum impurity decrease=0 | All | None |
| **Random forest** | 100 | Gini | Best nodes, minimum impurity decrease=0 | Square root | None |

**Supplementary table** **2**. **Hyperparameters for decision tree-based ML algorithms tested for GO term classification.**

|  |  | **RF classifier model** | | |
| --- | --- | --- | --- | --- |
| Gene Symbol | Rank | GSH | Mitochondria | Transporter |
| GSR | 1 | 1 | 0.9223 (0.0131) | 0.8558 (0.0203) |
| ETHE1 | 2 | 1 | 0.8648 (0.0425) | 0.8312 (0.0254) |
| PARK7 | 3 | 1 | 1 | 0.8120 (0.0092) |
| GLRX2 | 4 | 1 | 1 | 0.8252 (0.0134) |
| ALDH2 | 5 | 0.8809 (0.0136) | 1 | 0.8383 (0.0284) |
| IDH1 | 6 | 0.8778 (0.0195) | 0.8900 (0.0080) | 0.8440 (0.0171) |
| NENF | 7 | 0.8754 (0.0122) | 1 | 0.8042 (0.0353) |
| PC | 8 | 0.8638 (0.0141) | 0.9265 (0.0083) | 0.9315 (0.0180) |
| ABCB6 | 9 | 0.8542 (0.0127) | 0.9109 (0.0046) | 1 |
| OAT | 10 | 0.8496 (0.0110) | 0.9547 (0.0087) | 0.8755 (0.0146) |
| CPS1 | 11 | 0.8446 (0.0119) | 0.8516 (0.0289) | 0.8552 (0.0309) |
| SUCLG2 | 12 | 0.8405 (0.0110) | 0.9346 (0.0183) | 0.8648 (0.0222) |
| ACADVL | 13 | 0.8398 (0.0073) | 0.9245 (0.0256) | 0.8523 (0.0318) |
| MCU | 14 | 0.8314 (0.0072) | 1 | 1 |
| TIMM9 | 15 | 0.8199 (0.0082) | 0.9537 (0.0115) | 0.8742 (0.0215) |
| HSDL2 | 16 | 0.8185 (0.0346) | 0.9723 (0.0090) | 0.8964 (0.0442) |
| ATP5MC3 | 17 | 0.8166 (0.0363) | 1 | 0.8837 (0.0259) |
| ADH5 | 18 | 0.8128 (0.0237) | 1 | 1 |
| AIFM2 | 19 | 0.8118 (0.0095) | 1 | 0.9450 (0.0233) |
| PNKD | 20 | 0.8115 (0.0134) | 1 | 0.8403 (0.0609) |
| CHCHD5 | 21 | 0.8110 (0.0072) | 0.9744 (0.0145) | 0.9082 (0.0390) |
| HADHB | 22 | 0.8092 (0.0245) | 0.9384 (0.0100) | 0.8930 (0.0250) |
| CYB5A | 23 | 0.8089 (0.0425) | 0.9606 (0.0215) | 1 |
| ECHS1 | 24 | 0.8032 (0.0300) | 1 | 0.8652 (0.0370) |
| ATPAF1 | 25 | 0.8016 (0.0477) | 1 | 1 |
| MRPL22 | 26 | 0.8013 (0.0507) | 1 | 0.8844 (0.0345) |
| HKDC1 | 27 | 0.8003 (0.0424) | 1 | 0.8725 (0.0593) |

**Supplementary table** **3. Most probable non-SLC25 candidate mGSH transporters by mean RF classification probabilities with standard error, ranked by GSH probability**. Probability of 1 indicates the gene is already annotated by corresponding GO term.

|  |  | **Classifier** | |
| --- | --- | --- | --- |
| GO term | Classification threshold | DeepGOPlus | Random forest |
| GO:0006749 glutathione metabolic process | 0.4 | 0.053 | **0.850** |
|  | 0.5 | 0.026 | **0.800** |
|  | 0.6 | 0.000 | **0.650** |
| GO:0005739 mitochondrion | 0.4 | 0.507 | **0.932** |
|  | 0.5 | 0.320 | **0.887** |
|  | 0.6 | 0.033 | **0.777** |
| GO:0022857 transmembrane transporter activity | 0.4 | 0.727 | **0.966** |
|  | 0.5 | 0.593 | **0.894** |
|  | 0.6 | 0.387 | **0.771** |

**Supplementary table** **4.** **Sensitivity values for GO annotations by DeepGOPlus and hybrid RF (14 PC features) classifier models at different classification thresholds**. The best performing model for each annotation task at each classification threshold is bolded.

|  |  | **Metric** |  |
| --- | --- | --- | --- |
| Classifier model | MCC | AUROC | AUPRC |
| Glutamate metabolic process | 0.4835 | 0.8223 | 0.8110 |
| 2-oxoglutarate metabolic process | 0.5387 | 0.8224 | 0.8245 |
| Carnitine metabolic process | 0.4602 | 0.8107 | 0.8207 |

**Supplementary table 5. Model evaluations of non-GSH metabolite RF classifiers.** Values are mean metrics across feature sets (5-50 transcriptomic PCs). The GO term for classification is indicated by the “Classifier model” column.

| **Model information** | | | **Metric** | | | | |
| --- | --- | --- | --- | --- | --- | --- | --- |
| Tissue-type | Classification task | Dataset | Accuracy | Precision | Recall | MCC | F1-score |
| Whole transcriptomics | GSH | CCLE | 0.7570 | 0.7523 | 0.7951 | 0.5288 | 0.7644 |
|  |  | TCGA | 0.6568 | 0.6584 | 0.6853 | 0.3212 | 0.6605 |
|  | Mito | CCLE | 0.8197 | 0.7877 | 0.8767 | 0.6443 | 0.8294 |
|  |  | TCGA | 0.6902 | 0.7000 | 0.6687 | 0.3814 | 0.6834 |
|  | Transporter | CCLE | 0.7242 | 0.6889 | 0.8185 | 0.4581 | 0.7475 |
|  |  | TCGA | 0.6235 | 0.6180 | 0.6478 | 0.2482 | 0.6316 |
| Skin | Mito | CCLE | 0.8200 | 0.7915 | 0.8702 | 0.6440 | 0.8286 |
|  |  | TCGA | 0.6930 | 0.6881 | 0.7227 | 0.3872 | 0.7018 |
|  | Transporter | CCLE | 0.6272 | 0.6157 | 0.6793 | 0.2564 | 0.6453 |
|  |  | TCGA | 0.6068 | 0.5975 | 0.6567 | 0.2293 | 0.6250 |
| Liver | Mito | CCLE | 0.8177 | 0.7854 | 0.8758 | 0.6405 | 0.8278 |
|  |  | TCGA | 0.6978 | 0.6869 | 0.7208 | 0.3918 | 0.7006 |
|  | Transporter | CCLE | 0.6752 | 0.6557 | 0.7396 | 0.3542 | 0.6946 |
|  |  | TCGA | 0.6074 | 0.6015 | 0.6379 | 0.2224 | 0.6185 |
| Pancreas | Mito | CCLE | 0.8204 | 0.7894 | 0.8756 | 0.6459 | 0.8297 |
|  |  | TCGA | 0.6874 | 0.6775 | 0.7182 | 0.3762 | 0.6966 |
|  | Transporter | CCLE | 0.6542 | 0.6391 | 0.7103 | 0.3111 | 0.6722 |
|  |  | TCGA | 0.6039 | 0.5978 | 0.6371 | 0.2086 | 0.6162 |

**Supplementary table** **6.** **Performance metrics of RF classifiers in GO term annotation tasks using disease-specific CCLE or TCGA transcriptomics features**. Except for whole transcriptomics models, the classifies are trained and tested on samples from a single source tissue (skin, liver, or pancreas). Metrics are mean values for RF classifiers using only 7, 16 and 32 transcriptomics PCs as features (Due to limited number of CCLE liver samples, a maximum of 24 PC features are used in these classifiers). Whole transcriptomics classifiers include 52 PC feature models.

**
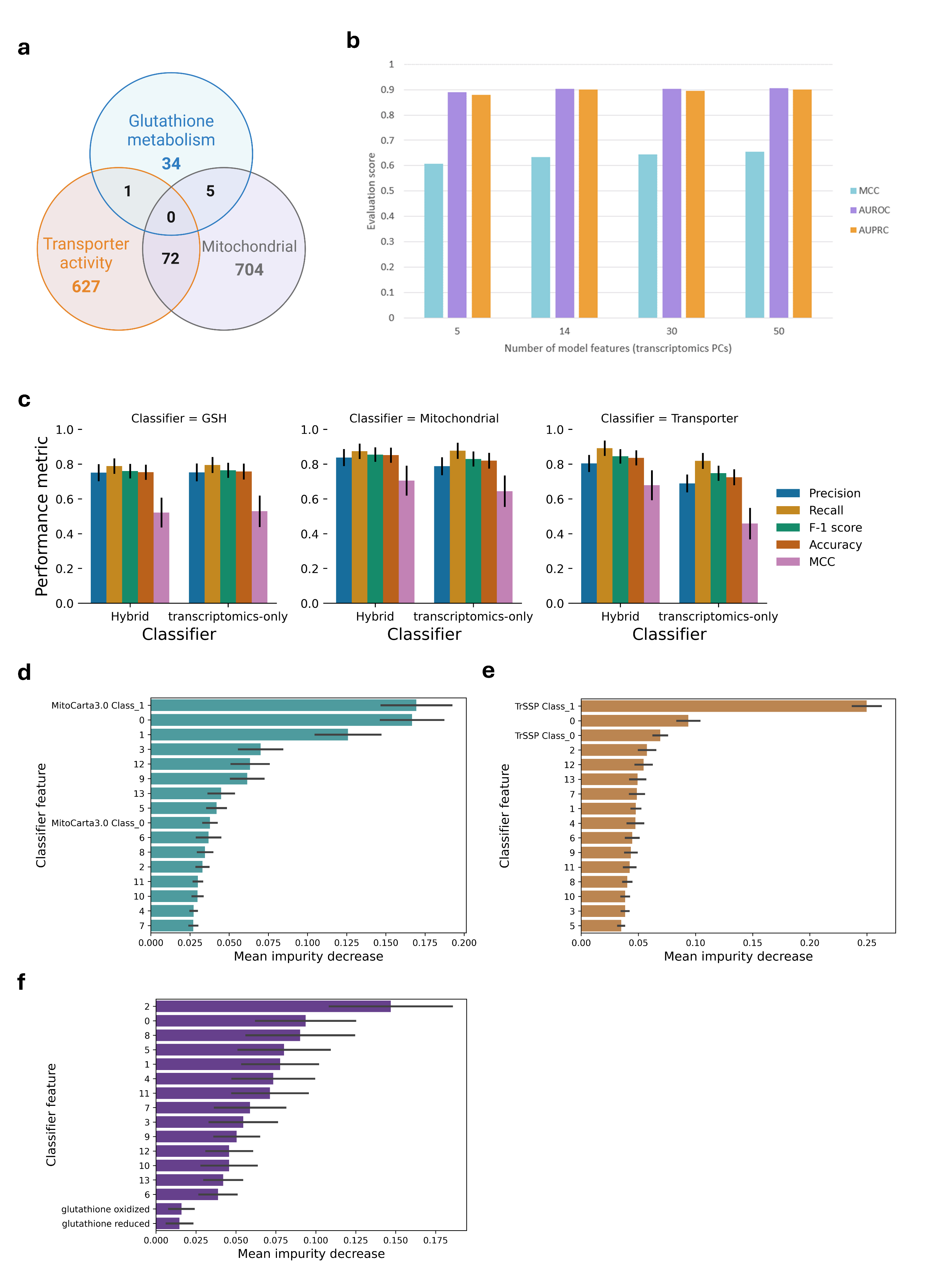
Supplementary Figure 1.** Evaluation of mitochondrial, glutathione, and transporter GO terms and classifiers used in to identify candidate mGSH transporters. (**a**) Overlap in genes annotated with GO terms used in classifier training and testing. (**b**) Mean RF classifier evaluation metric scores with different numbers of principal component features. (**c**) Evaluation of hybrid and transcriptomics-only classifiers. Error bars indicate standard deviation of mean values across classifiers over bootstrap iterations and different numbers of principal component feature sets (5-50 or 7-52 for transcriptomics-only model). (**d-f**) Mean RF classifier feature importance as measured by mean impurity decrease across trees for mitochondrial (**d**), transporter activity (**e**), and GSH metabolism (**f**) GO term classifiers. Feature importance values are averages of RF classifiers with 5, 14, 30, and 50 PC features. Error bars are standard deviations.


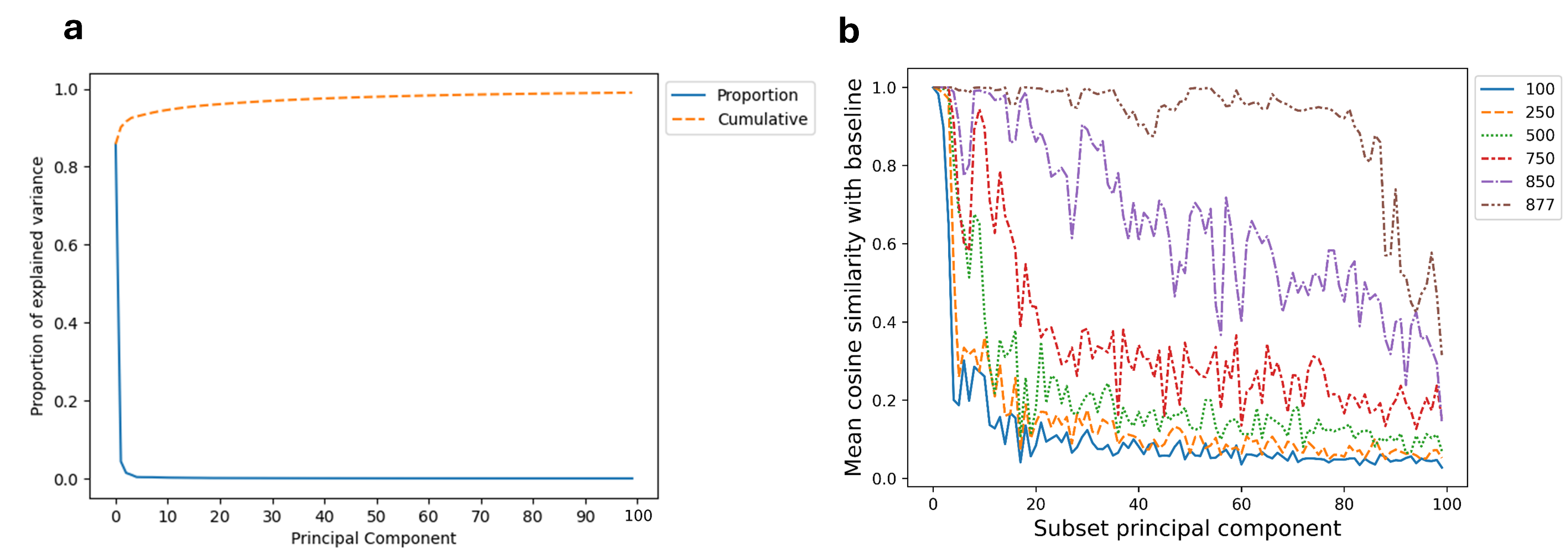


**Supplementary Figure** **2**. (**a**) Explained variance by principal components generated from whole CCLE transcriptomics data. Cumulative explained variance for the first *n* principal components (orange, dashed) and explained variance proportion per component (blue, solid) are shown. (b) Mean cosine similarity between random subset principal components and baseline components computed from whole CCLE transcriptomics data. Subset principal components are first aligned to baseline components. 20 random subsets are generated for each sample size.


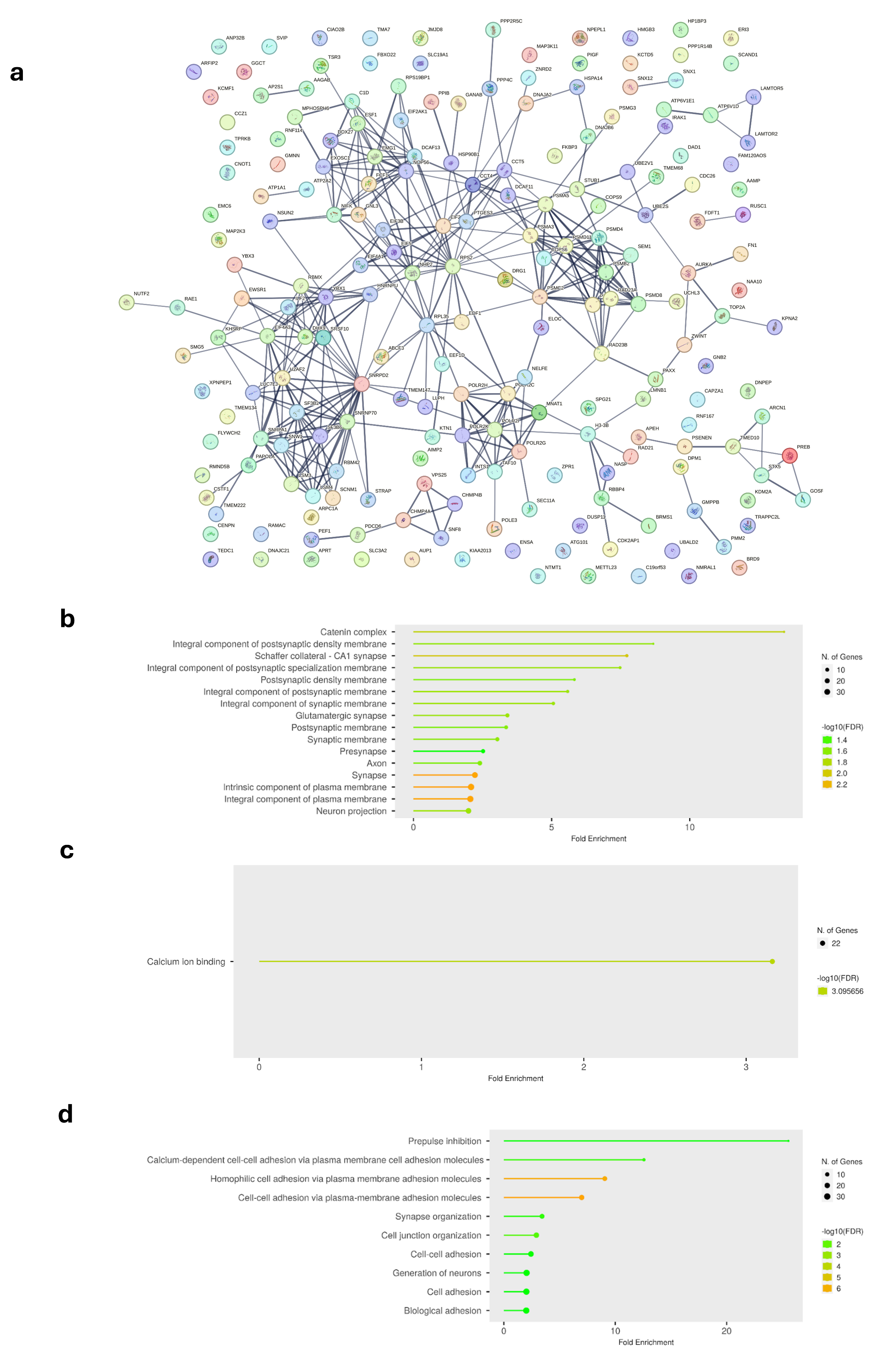


**Supplementary figure** **3**. Analysis of false positive genes by mitochondrial and transporter classifiers. (**a**) STRING network of 206 genes predicted to localize to mitochondria by RF classifiers with no existing evidence in mitochondria databases. Strength of data support for connection is indicated by edge boldness (minimum confidence score of 0.70). 61 nodes with no edges, 86 nodes with no connection to the central network. (**b-**d) GO enrichment analysis of genes predicted to have transmembrane transporter activity by RF classifiers with no existing evidence in transporter databases. Enrichment plots of false positive transporter genes for cellular component (**b**), molecular function (**c**), and biological process (**d**), GO terms.

**
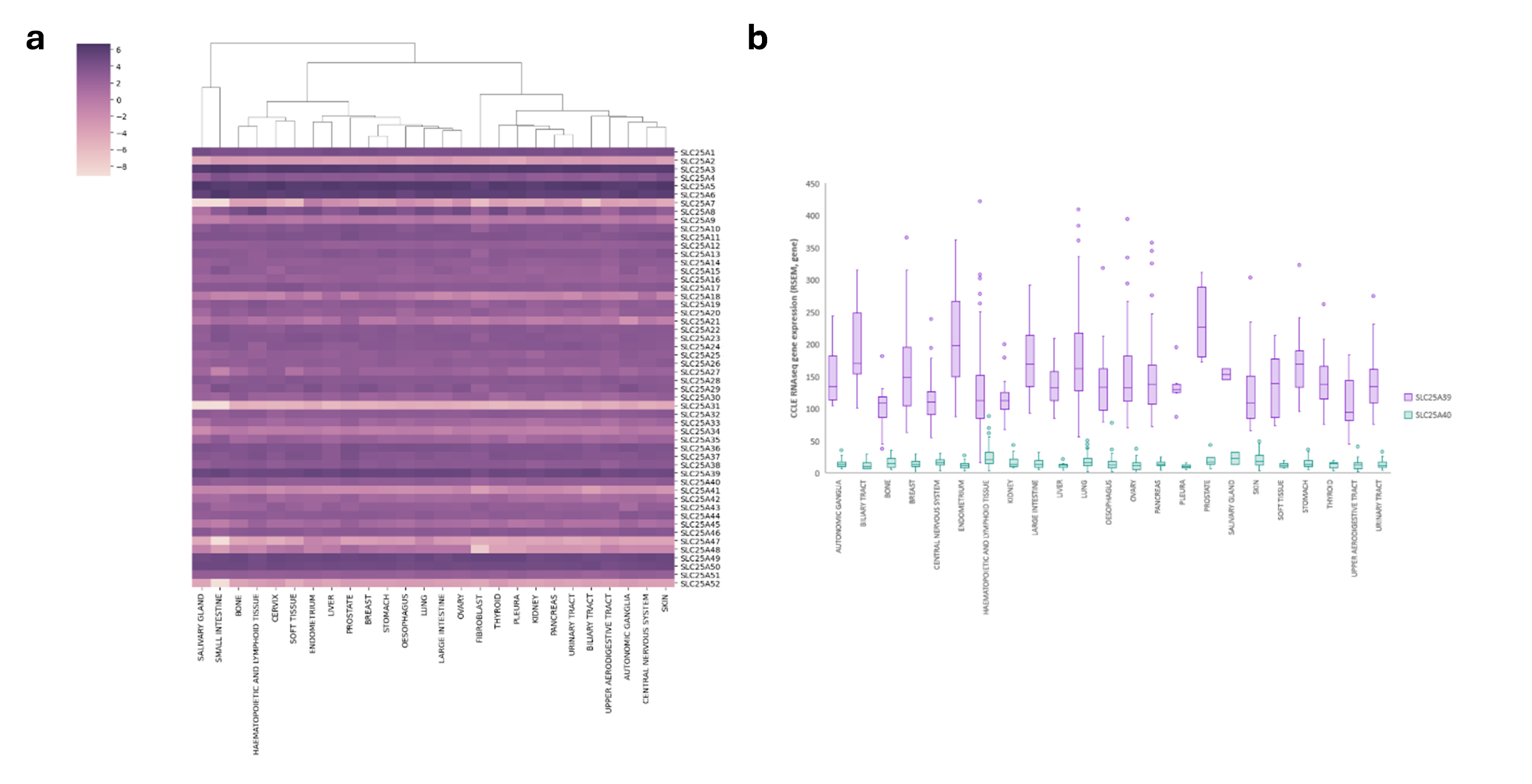
**

**Supplementary Figure 4.** CCLE transcriptomics of SLC25 family members. (**a**) Mean SLC25 gene expression (log2 RNA-Seq by Expectation Maximization (RSEM)) across CCLE transcriptomics tissue types. Tissues are clustered using agglomerative clustering through ward linkages of euclidean distances. (**b**) TPM values across CCLE tissue types for known GSH transporters SLC25A39 (purple) and SLC25A40 (green).


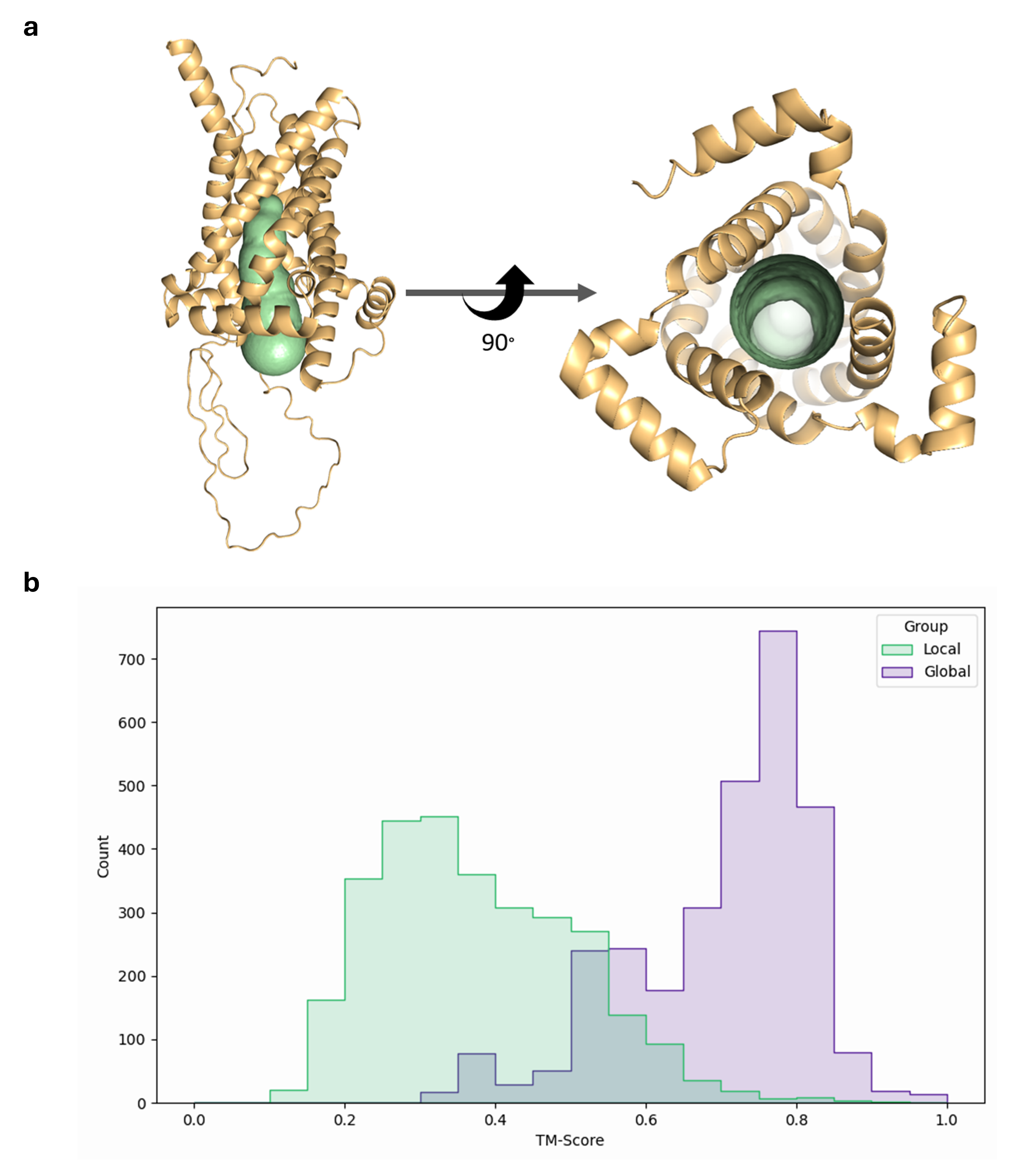


**Supplementary Figure 5.** Structural analysis of CAVER identified transport tunnel residues in SLC25 structures. (**a**) CAVER tunnel residue surface indicated in green, these residues are then used for local TM-alignments to identify similarities in substrate binding and transport function. Protein structure is AlphaFold-predicted SLC25A39 structure. (**b**) Distribution of alignment scores by TM-align for pairs of SLC25 structures using entire protein structures (“Global”, purple) or tunnel residues only (“Local”, green) to calculate alignment.


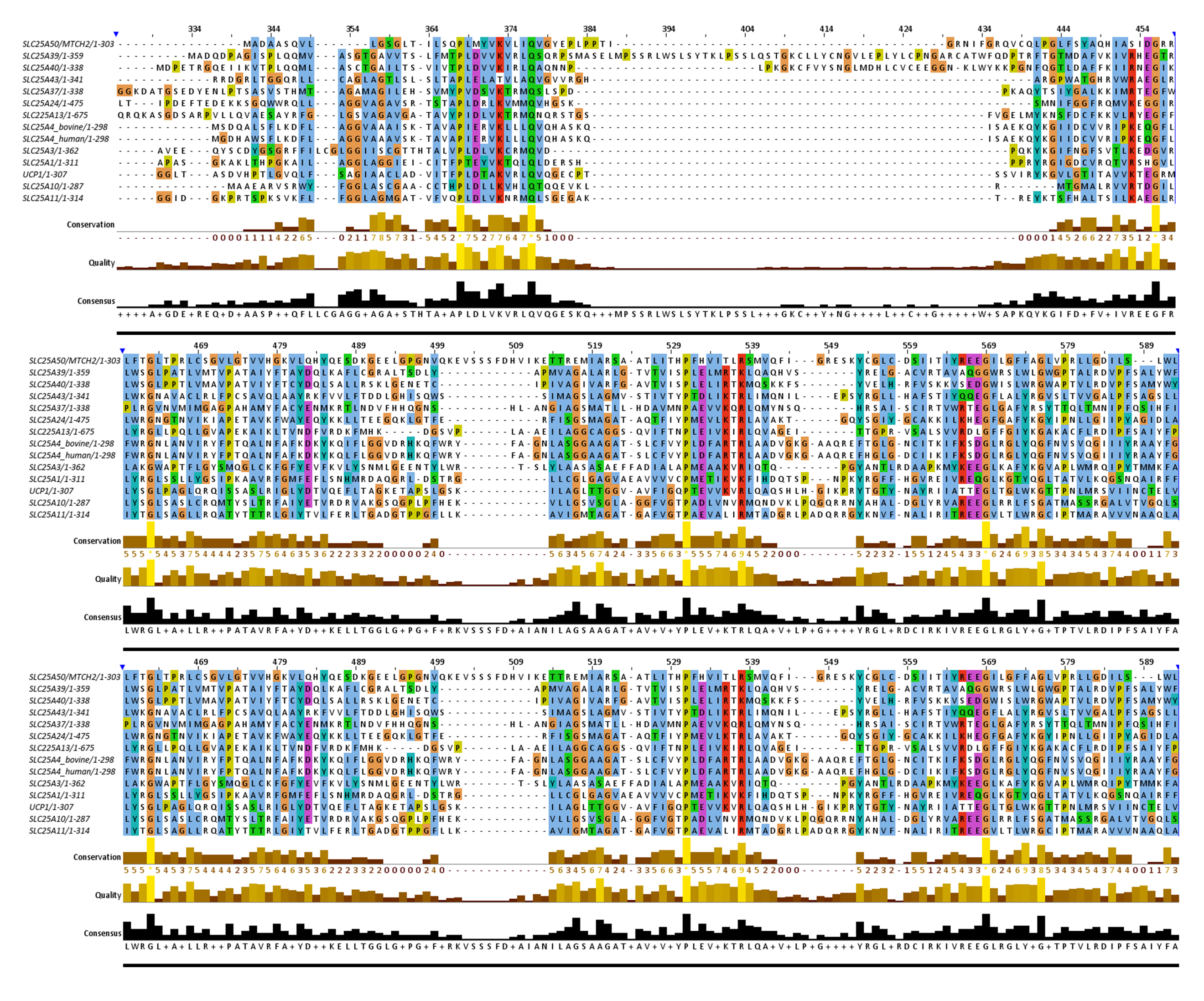
 **Supplementary Figure 6**. Multiple sequence alignment of SLC25 sequences used in structural experiments. Names of genes for each aligned sequence are indicated. Aligned regions of the amino acid sequences are shown with residues coloured according to their properties. Alignment conservation, quality, and consensus at each position are indicated beneath the multiple sequence alignments.


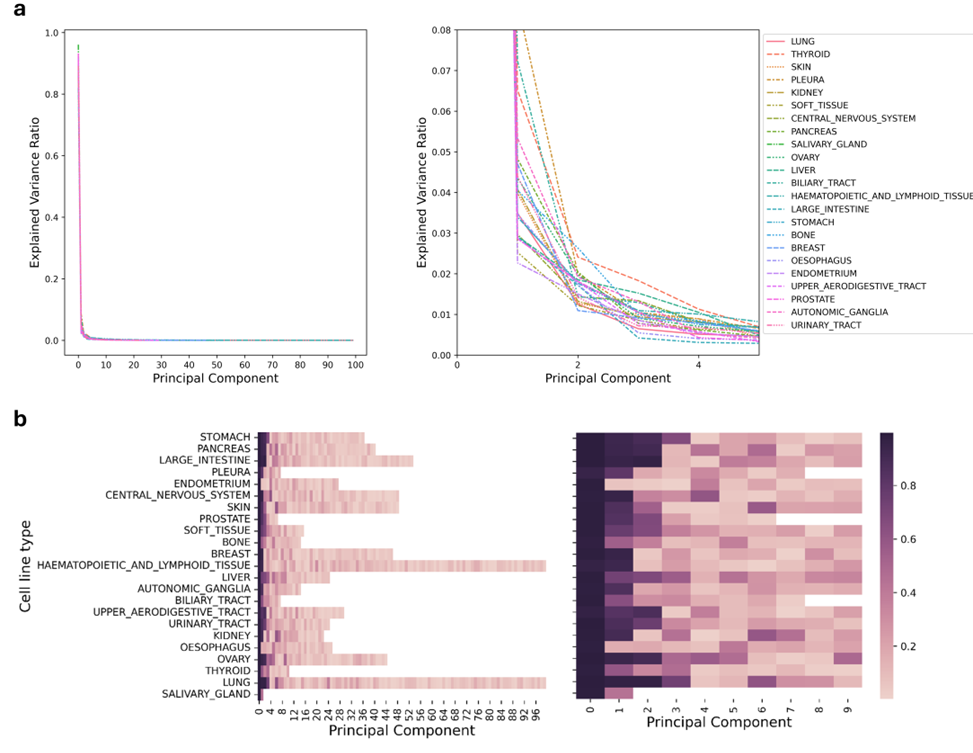


**Supplementary Figure 7**. Differences across CCLE tissue-types through PCA. (**a**) Scree plot of explained variance ratios by tissue-type specific principal components from CCLE transcriptomics data. (b) Cosine similarity between tissue-type specific principal components and baseline components computed from whole CCLE transcriptomics data. Tissue-type principal components are first aligned to baseline components.

**
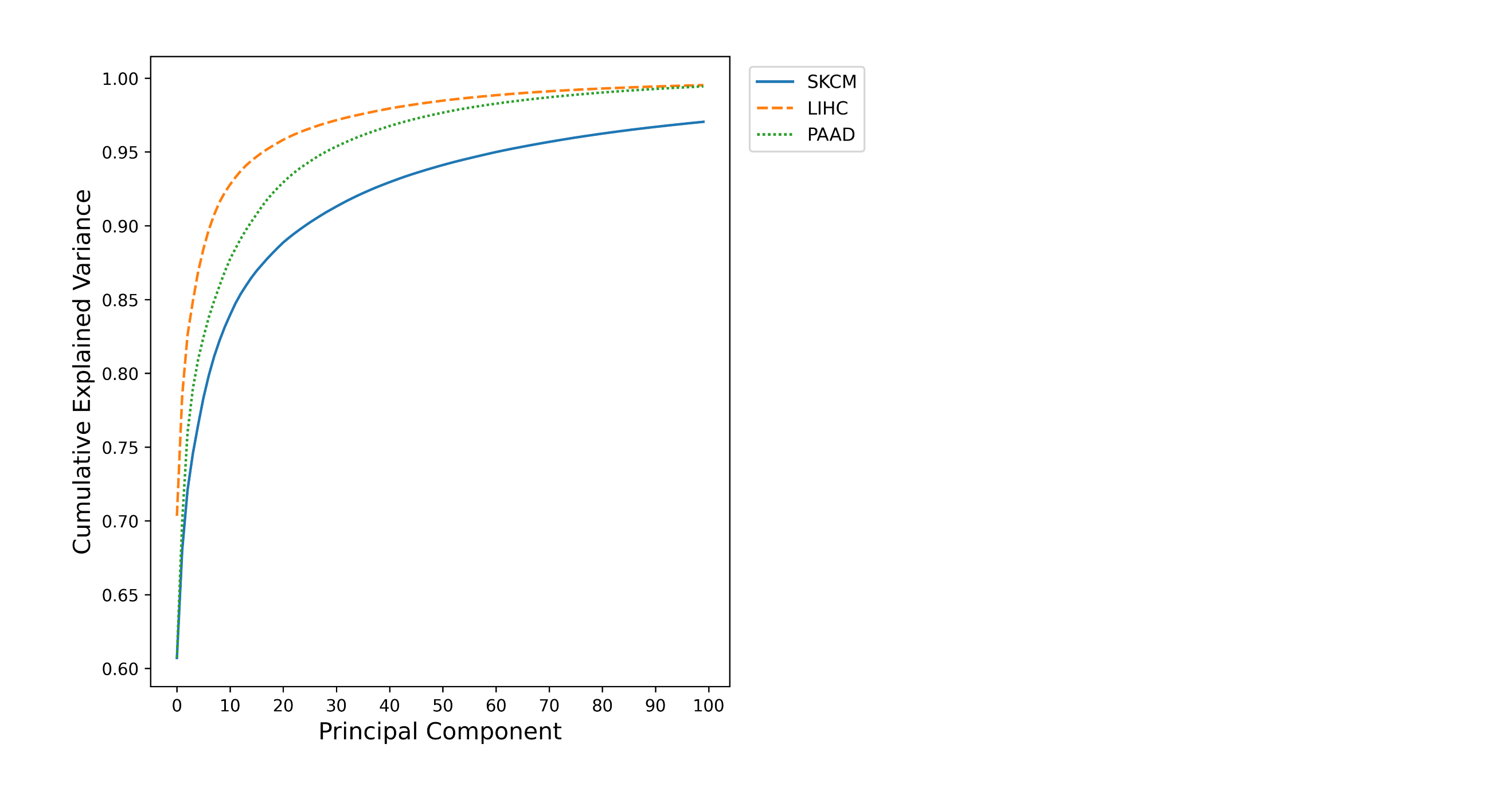
Supplementary Figure 8.** Cumulative explained variance by principal components for gene expression values of TCGA primary tumor samples for skin cutaneous melanoma (SKCM), liver hepatocellular carcinoma (LIHC) and pancreatic adenocarcinoma (PAAD).

**
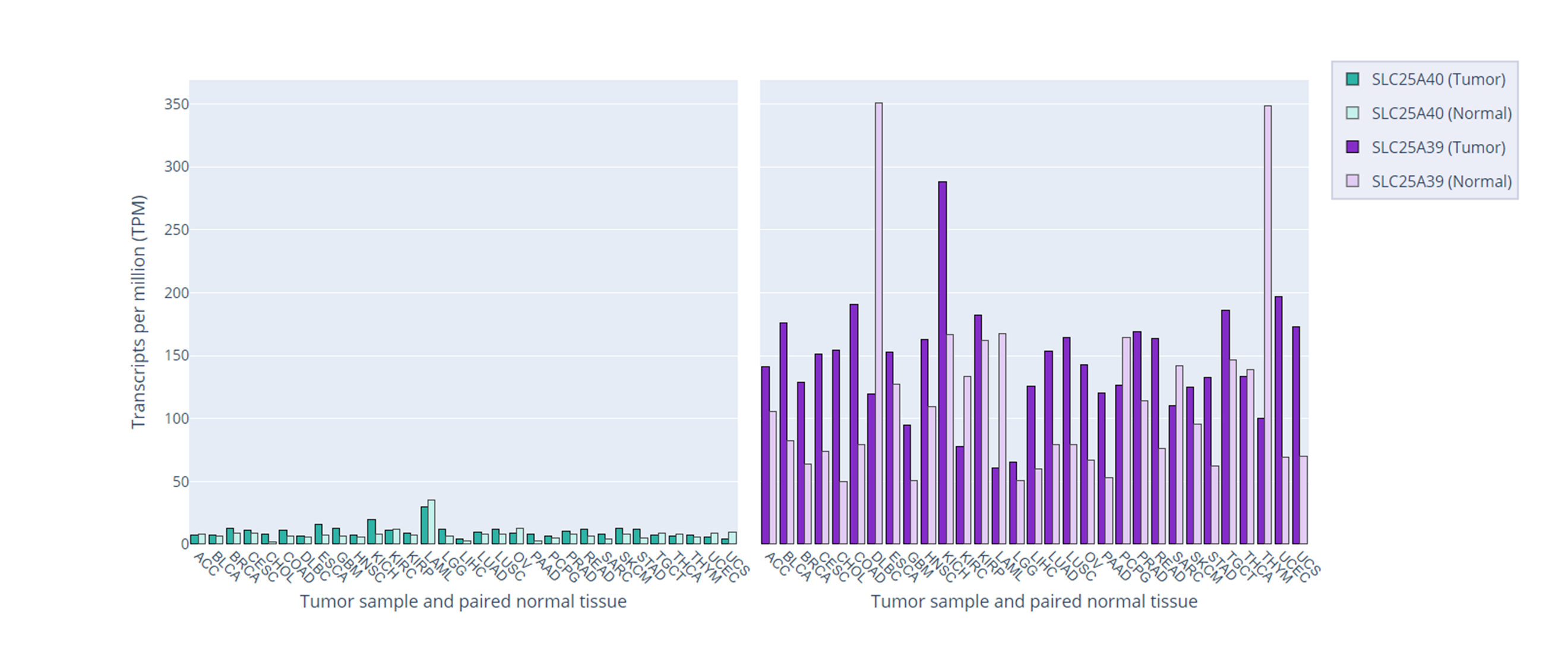
**

**Supplementary Figure 9.** Median TPM values across paired TCGA tumor and GTEx normal tissue samples for SLC25A39 (purple) and SLC25A40 (green). Expression data accessed via GEPIA.
